# Impact of Network Structure and Cellular Response on Spike Time Correlations

**DOI:** 10.1371/journal.pcbi.1002408

**Published:** 2012-03-22

**Authors:** James Trousdale, Yu Hu, Eric Shea-Brown, Krešimir Josić

**Affiliations:** 1 Department of Mathematics, University of Houston, Houston, Texas, United States of America; 2 Department of Applied Mathematics, Program in Neurobiology and Behavior, University of Washington, Seattle, Washington, United States of America; 3 Department of Biology and Biochemistry, University of Houston, Houston, Texas, United States of America; Indiana University, United States of America

## Abstract

Novel experimental techniques reveal the simultaneous activity of larger and larger numbers of neurons. As a result there is increasing interest in the structure of cooperative – or *correlated* – activity in neural populations, and in the possible impact of such correlations on the neural code. A fundamental theoretical challenge is to understand how the architecture of network connectivity along with the dynamical properties of single cells shape the magnitude and timescale of correlations. We provide a general approach to this problem by extending prior techniques based on *linear response theory*. We consider networks of general integrate-and-fire cells with arbitrary architecture, and provide explicit expressions for the approximate cross-correlation between constituent cells. These correlations depend strongly on the operating point (input mean and variance) of the neurons, even when connectivity is fixed. Moreover, the approximations admit an expansion in powers of the matrices that describe the network architecture. This expansion can be readily interpreted in terms of paths between different cells. We apply our results to large excitatory-inhibitory networks, and demonstrate first how precise *balance* – or lack thereof – between the strengths and timescales of excitatory and inhibitory synapses is reflected in the overall correlation structure of the network. We then derive explicit expressions for the average correlation structure in randomly connected networks. These expressions help to identify the important factors that shape coordinated neural activity in such networks.

## Author Summary

Is neural activity more than the sum of its individual parts? What is the impact of cooperative, or *correlated*, spiking among multiple cells? We can start addressing these questions, as rapid advances in experimental techniques allow simultaneous recordings from ever-increasing populations. However, we still lack a general understanding of the origin and consequences of the joint activity that is revealed. The challenge is compounded by the fact that both the intrinsic dynamics of single cells and the correlations among then vary depending on the overall state of the network. Here, we develop a toolbox that addresses this issue. Specifically, we show how *linear response theory* allows for the expression of correlations explicitly in terms of the underlying network connectivity and known single-cell properties – and that the predictions of this theory accurately match simulations of a touchstone, nonlinear model in computational neuroscience, the general integrate-and-fire cell. Thus, our theory should help unlock the relationship between network architecture, single-cell dynamics, and correlated activity in diverse neural circuits.

## Introduction

New multielectrode and imaging techniques are revealing the simultaneous activity of neural ensembles and, in some cases, entire neural populations [1]–[4]. This has thrust upon the computational biology community the challenge of characterizing a potentially complex set of interactions – or *correlations* – among pairs and groups of neurons.

Beyond important and rich challenges for statistical modeling [5], the emerging data promises new perspectives on the neural encoding of information [6]. The structure of correlations in the activity of neuronal populations is of central importance in understanding the neural code [7]–[13]. However, theoretical [9]–[11], [14]–[16], and empirical studies [17]–[19] do not provide a consistent set of general principles about the impact of correlated activity. This is largely because the presence of correlations can either strongly increase or decrease the fidelity of encoded information depending on both the structure of correlations across a population and how their impact is assessed.

A basic mechanistic question underlies the investigation of the role of collective activity in coding and signal transmission: How do single-cell dynamics, connection architecture, and synaptic dynamics combine to determine patterns of network activity? Systematic answers to this question would allow us to predict how empirical data from one class of stimuli will generalize to other stimulus classes and recording sites. Moreover, a mechanistic understanding of the origin of correlations, and knowledge of the patterns we can expect to see under different assumptions about the underlying networks, will help resolve recent controversies about the strength and pattern of correlations in mammalian cortex [1], [20], [21]. Finally, understanding the origin of correlations will inform the more ambitious aim of inferring properties of network architecture from observed patterns of activity [22]–[24].

Here, we examine the link between network properties and correlated activity. We develop a theoretical framework that accurately predicts the structure of correlated spiking that emerges in a widely used model – recurrent networks of general integrate and fire cells. The theory naturally captures the role of single cell and synaptic dynamics in shaping the magnitude and timescale of spiking correlations. We focus on the exponential integrate and fire model, which has been shown to capture membrane and spike responses of cortical neurons [25]; however, the general approach we take can be applied to a much broader class of neurons, a point we return to in the Discussion.

Our approach is based on an extension of linear response theory to networks [24], [26]. We start with a linear approximation of a neuron's response to an input. This approximation can be obtained explicitly for many neuron models [27]–[29], and is directly related to the spike triggered average [30]. The correlation structure of the network is then estimated using an iterative approach. As in prior work [31]–[33], the resulting expressions admit an expansion in terms of paths through the network.

We apply this theory to networks with precisely balanced inhibition and excitation in the inputs to individual cells. In this state individual cells receive a combination of excitatory and inhibitory inputs with mean values that largely cancel. We show that, when timescales and strengths of excitatory and inhibitory connections are matched, only local interactions between cells contribute to correlations. Moreover, our theory allows us to explain how correlations are altered when precise tuning balance is broken. In particular, we show how strengthening inhibition may synchronize the spiking activity in the network. Finally, we derive results which allow us to gain an intuitive understanding of the factors shaping average correlation structure in randomly connected networks of neurons.

## Results

Our goal is to understand how the architecture of a network shapes the statistics of its activity. We show how correlations between spike trains of cells can be approximated using response characteristics of individual cells along with information about synaptic dynamics, and the structure of the network. We start by briefly reviewing linear response theory of neuronal responses [28], [34], [35], and then use it to approximate the correlation structure of a network.

### Network model

To illustrate the results we consider a network of 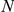 nonlinear integrate-and-fire (IF) neurons with membrane potentials modeled by
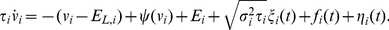
(1)Here 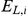 is the leak reversal potential, and 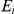 represents the mean synaptic input current from parts of the system not explicitly modeled. A spike-generating current 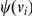 may be included to emulate the rapid onset of action potentials. Unless otherwise specified, we utilize the exponential IF model (EIF), so that 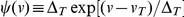 [25]. Cells are subject to internally induced fluctuations due to channel noise [36], and externally induced fluctuations due to inputs not explicitly modelled [37]. We model both by independent, Gaussian, white noise processes, 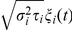 [38]. An external signal to cell 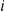 is represented by 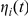.

Upon reaching a threshold 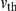, an action potential is generated, and the membrane potential is reset to 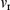, where it is held constant for an absolute refractory period 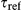. The output of cell 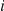 is characterized by the times, 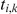, at which its membrane potential reaches threshold, resulting in an output spike train 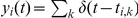. Synaptic interactions are modeled by delayed 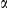-functions
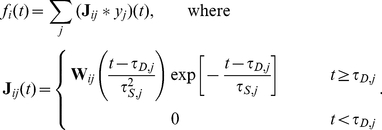
(2)The 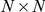 matrix 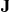 contains the synaptic kernels, while the matrix 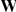 contains the synaptic weights, and hence defines the network architecture. In particular, if 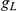 is the membrane conductance, 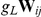 is the area under a post-synaptic current evoked in cell 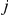 by a spike in the presynaptic cell 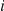, and along with the membrane and synaptic time constants, determines the area under a post-synaptic potential. 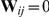 represents the absence of a synaptic connection from cell 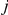 to cell 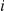.

Table 1 provides an overview of all parameters and variables.

**Table 1:** Notation used in the text.

Symbol	Description
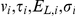	Membrane potential, membrane time constant, leak reversal potential, and noise intensity of cell 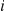 .
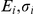	Mean and standard deviation of the background noise for cell 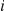 .
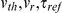	Membrane potential threshold, reset, and absolute refractory period for cells.
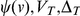	Spike generating current, soft threshold and spike shape parameters for the IF model [25].
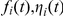	Synaptic input from other cells in the network, and external input to cell 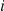 .
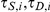	Synaptic time constant and delay for outputs of cell 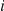 .
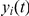	Spike train of cell 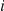 .
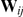	The 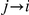 synaptic weight, proportional to the area under a single post-synaptic current for current-based synapses.
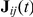	The 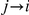 synaptic kernel - equals the product of the synaptic weight 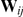 and the synaptic filter for outputs of cell 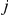 .
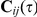	The cross-correlation function between cells 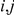 defined by 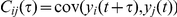 .
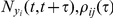	Spike count for cell 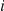 , and spike count correlation coefficient for cells 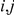 over windows of length 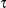 .
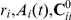	Stationary rate, linear response kernel and uncoupled auto-correlation function for cell 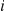 j.
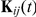	The 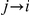 interaction kernel - describes how the firing activity of cell 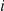 is perturbed by an input spike from cell 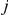 . It is defined by 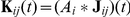 .
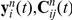	The 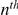 order approximation of the activity of cell 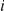 in a network which accounts for directed paths through the network graph up to length 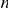 ending at cell 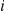 , and the cross-correlation between the 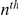 order approximations of the activity of cells 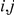 .
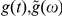	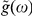 is the Fourier transform of 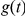 with the convention
	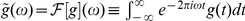

### Linear response of individual cells

Neuronal network models are typically described by a complex system of coupled nonlinear stochastic differential equations. Their behavior is therefore difficult to analyze directly. We will use linear response theory [28], [34], [35], [39] to approximate the cross-correlations between the outputs of neurons in a network. We first review the linear approximation to the response of a single cell. We illustrate the approach using current-based IF neurons, and explain how it can be generalized to other models in the Discussion.

**Figure 1:**
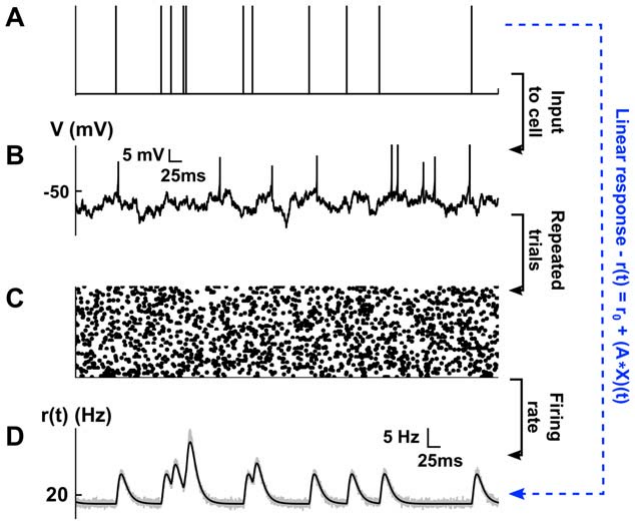
Illustrating **Eq. (4)**. (**A**) The input to the post-synaptic cell is a fixed spike train which is convolved with a synaptic kernel. (**B**) A sample voltage path for the post-synaptic cell receiving the input shown in A) in the presence of background noise. (**C**) Raster plot of 100 realizations of output spike trains of the post-synaptic cell. (**D**) The output firing rate, 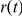, obtained by averaging over realizations of the output spike trains in C). The rate obtained using Monte Carlo simulations (shaded in gray) matches predictions of linear response theory obtained using Eq. (4) (black).

The membrane potential of an IF neuron receiving input 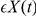, with vanishing temporal average, 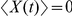, evolves according to
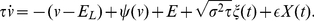
(3)The time-dependent firing rate, 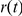, is determined by averaging the resulting spike train, 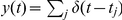, across different realizations of noise, 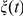, for fixed 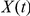. Using linear response theory, we can approximate the firing rate by
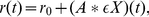
(4)where 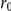 is the (stationary) firing rate when 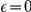. The linear response kernel, 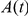, characterizes the firing rate response to first order in 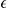. A rescaling of the function 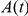 gives the spike-triggered average of the cell, to first order in input strength, and is hence equivalent to the optimal Weiner kernel in the presence of the signal 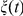. [39], [40]. In Figure 1, we compare the approximate firing rate obtained from Eq. (4) to that obtained numerically from Monte Carlo simulations.

The linear response kernel 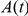 depends implicitly on model parameters, but is independent of the input signal, 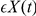, when 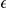 is small relative to the noise 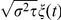. In particular, 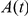 is sensitive to the value of the mean input current, 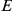. We emphasize that the presence of the background noise, 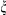, in Eq. (3) is essential to the theory, as noise linearizes the transfer function that maps input to output. In addition, when applying linear response methods, there is an implicit assumption that the fluctuations of the input 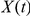 do not have a significant effect on the response properties of the cell.

### Linear response in recurrent networks

The linear response kernel can be used to approximate the response of a cell to an external input. However, the situation is more complicated in a network where a neuron can affect its own activity through recurrent connections. To extend the linear response approximation to networks we follow the approach introduced by Lindner et al. [26]. Instead of using the linear response kernel to approximate the firing rate of a cell, we use it to approximate a realization of its output
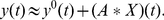
(5)Here 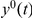 represents a realization of the spike train generated by an integrate-and-fire neuron obeying Eq. (3) with 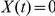.

Our central assumption is that a cell acts approximately as a linear filter of its inputs. Note that Eq. (5) defines a mixed point and continuous process, but averaging 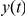 in Eq. (5) over realizations of 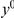 leads to the approximation in Eq. (4). Hence, Eq. (5) is a natural generalization of Eq. (4) with the unperturbed output of the cell represented by the point process, 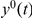, instead of the firing rate, 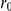.

We first use Eq. (5) to describe spontaneously evolving networks where 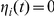. Equation (1) can then be rewritten as
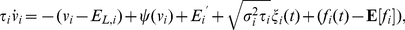
(6)where 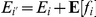 and 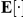 represents the temporal average.

Lindner et al. used Eq. (5) as an ansatz to study the response of an all–to–all inhibitory network. They postulated that the spiking output 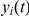 of cell 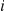 in the network, can be approximated in the frequency domain by
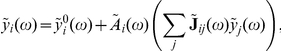
where 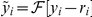 are the zero-mean Fourier transforms of the processes 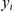, and 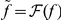 for all other quantities (see Table 1 for the Fourier transform convention). The term in parentheses is the Fourier transform of the zero-mean synaptic input, 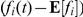, in Eq. (6), and 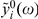 represents a realization of the spiking output of cell 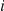 in the absence of synaptic fluctuations from the recurrent network (*i.e* assuming 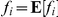). In matrix form this ansatz yields a simple self-consistent approximation for the firing activities 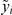 which can be solved to give
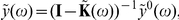
where the interaction matrix 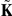 has entries defined by 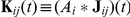. When averaged against its conjugate transpose, this expression yields an approximation to the full array of cross-spectra in the recurrent network:
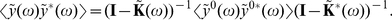
(7)


We next present a distinct derivation of this approximation which allows for a different interpretation of the ansatz given by Eq. (5). We iteratively build to the approximation in Eq. (7), showing how this expression for the correlation structure in a recurrent network can be obtained by taking into account the paths through the network of increasing length.

We start with realizations of spike trains, 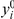, generated by IF neurons obeying Eq. (6) with 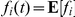. This is equivalent to considering neurons isolated from the network, with adjusted DC inputs (due to mean network interactions). Following the approximation given by Eq. (5), we use a *frozen* realization of all 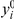 to find a correction to the output of each cell, with 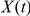 set to the mean-adjusted synaptic input,
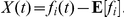
As noted previously, the linear response kernel is sensitive to changes in the mean input current. It is therefore important to include the average synaptic input 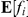 in the definition of the effective mean input, 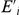.

The input from cell 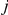 to cell 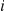 is filtered by the synaptic kernel 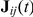. The linear response of cell 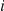 to a spike in cell 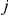 is therefore captured by the interaction kernel 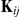, defined above as
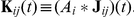
The output of cell 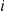 in response to mean-adjusted input, 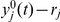, from cell 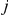 can be approximated to first order in input strength using the linear response correction
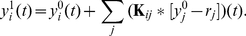
(8)We explain how to approximate the stationary rates, 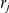, in the Methods.

The cross-correlation between the processes 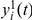 in Eq. (8) gives a first approximation to the cross-correlation function between the cells,
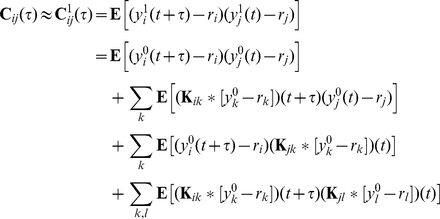
which can be simplified to give
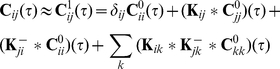
(9)where we used 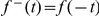. Ostojic et al. obtained an approximation closely related to Eq. (9). [24] They first obtained the cross-correlation between a pair of neurons which either receive a common input *or* share a monosynaptic connection. This can be done using Eq. (4), without the need to introduce the mixed process given in Eq. (5). Ostojic et al. then implicitly assumed that the correlations not due to one of these two submotifs could be disregarded. The correlation between pairs of cells which were mutually coupled (or were unidirectionally coupled with common input) was approximated by the sum of correlations introduced by each submotif individually.

Equation (9) provides a first approximation to the joint spiking statistics of cells in a recurrent network. However, it captures only the effects of direct synaptic connections, represented by the second and third terms, and common input, represented by the last term in Eq. (9). The impact of larger network structures, such as loops and chains are not captured, although they may significantly impact cross-correlations [41]–[43]. Experimental studies have also shown that local cortical connectivity may not be fully random [44]–[46]. It is therefore important to understand the effects on network architecture on correlations.

We therefore propose an iterative approach which accounts for successively larger connectivity patterns in the network [32], [33]. We again start with 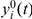, a realization of a single spike train in isolation. Successive approximations to the output of cells in a recurrent network are defined by
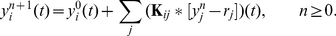
(10)


**Figure 2:**
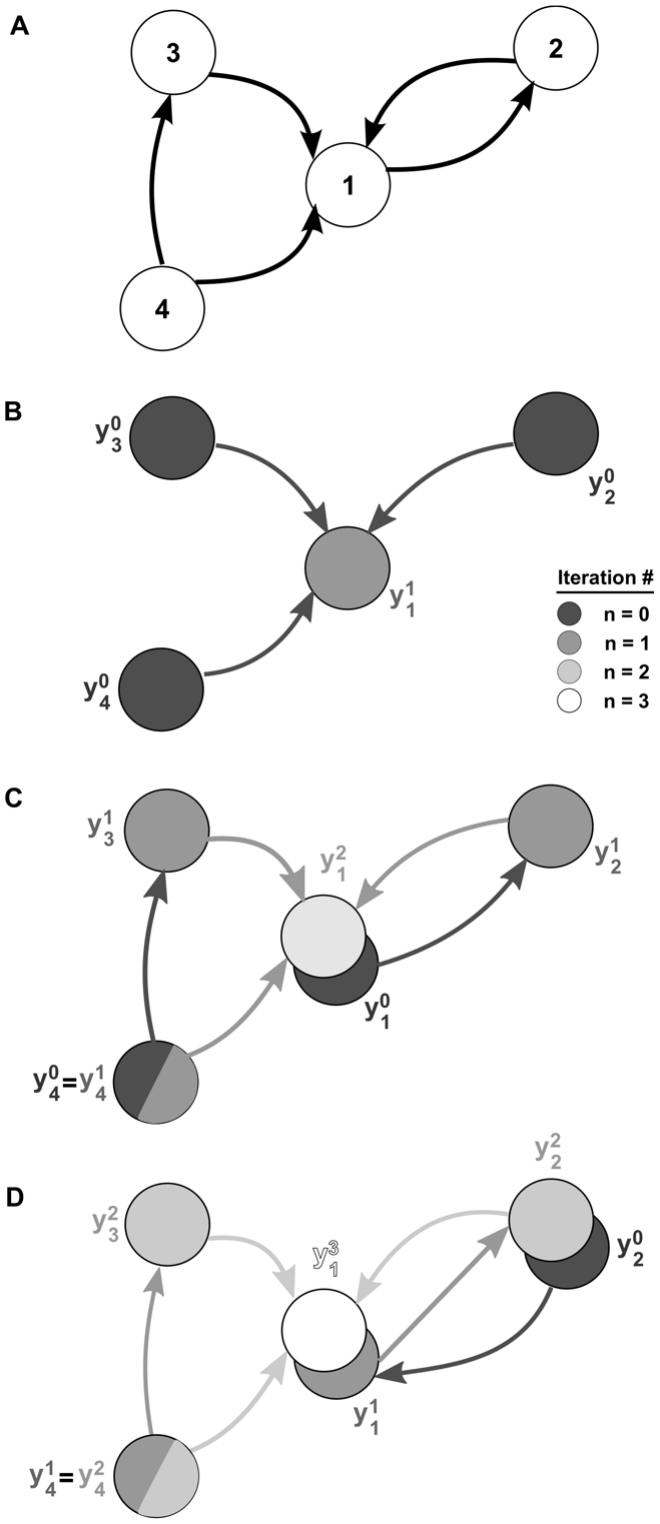
Iterative construction of the linear approximation to network activity. (**A**) An example recurrent network. (**B**)–(**D**) A sequence of graphs determines the successive approximations to the output of neuron 1. Processes defined by the same iteration of Eq. (11) have equal color. (**B**) In the first iteration of Eq. (11), the output of neuron 1 is approximated using the unperturbed outputs of its neighbors. (**C**) In the second iteration the results of the first iteration are used to define the inputs to the neuron. For instance, the process 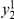 depends on the base process 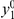 which represents the unperturbed output of neuron 1. Neuron 4 receives no inputs from the rest of the network, and all approximations involve only its unperturbed output, 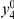. (**D**) Cells 3 and 4 are not part of recurrent paths, and their contributions to the approximation are fixed after the second iteration. However, the recurrent connection between cells 1 and 2 implies that subsequent approximations involve contributions of directed chains of increasing length.

To compute the correction to the output of a neuron, in the first iteration we assume that its inputs come from a collection of isolated cells: When 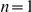, Eq. (10) takes into account only inputs from immediate neighbors, treating each as disconnected from the rest of the network. The corrections in the second iteration are computed using the approximate cell responses obtained from the first iteration. Thus, with 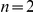, Eq. (10) also accounts for the impact of next nearest neighbors. Successive iterations include the impact of directed chains of increasing length: The isolated output from an independent collection of neurons is filtered through 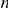 stages to produce the corrected response (See Figure 2.)

Notation is simplified when this iterative construction is recast in matrix form to obtain
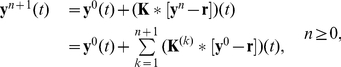
(11)where 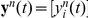 and 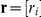 are length 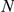 column vectors, and 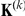 represents a 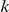-fold matrix convolution of 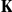 with itself. We define the convolution of matrices in the Methods.

The 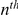 approximation to the matrix of cross-correlations can be written in terms of the interaction kernels, 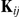, and the autocorrelations of the base processes 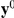 as
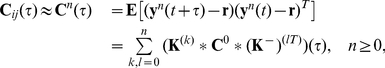
(12)where 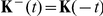, 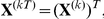 and 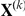 is the 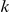-fold matrix convolution of 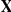 with itself.

Eq. (12) can be verified by a simple calculation. First, Eq. (11) directly implies that
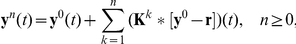
which we may use to find, for each 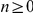,
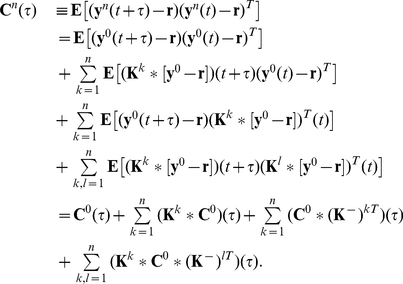
(13)Since 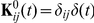, Eq. (13) is equivalent to Eq. (12).

If we apply the Fourier transform, to Eq. (12), we find that for each 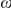,
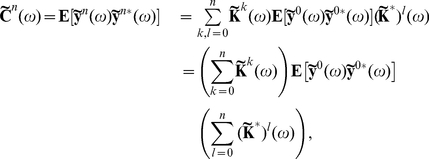
(14)where 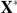 denotes the conjugate transpose of the matrix 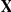. As before, the zero-mean Fourier transforms 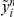 of the processes 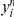 are defined by 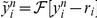, and 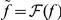 for all other quantities.

Defining 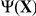 to be the spectral radius of the matrix 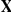, when 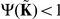, we can take the limit 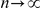 in Eq. (14) [47], [48], to obtain an approximation to the full array of cross-spectra
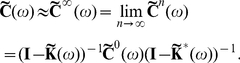
(15)As noted previously, this generalizes the approach of Lindner et al. [26] (also see [13]). In the limit 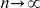, directed paths of arbitrary length contribute to the approximation. Equation (15) therefore takes into account the full recurrent structure of the network. Note that Eq. (15) may be valid even when 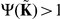. However, in this case the series in Eq. (14) do not converge, and hence the expansion of the correlations in terms of paths through the network is invalid. We confirmed numerically that 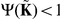 for all of the networks and parameters we considered.

Finally, consider the network response to external signals, 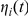, with zero mean and finite variance. The response of the neurons in the recurrent network can be approximated iteratively by
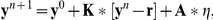
where 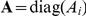 and 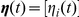. External signals and recurrent synaptic inputs are both linearly filtered to approximate a cell's response, consistent with a generalization of Eq. (4). As in Eq. (12), the 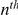 approximation to the matrix of correlations is
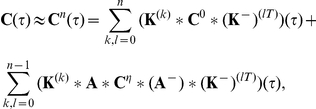
where 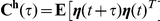 is the covariance matrix of the external signals. We can again take the Fourier transform and the limit 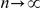, and solve for 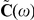. If 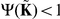,
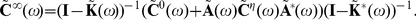
(16)When the signals comprising 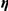 are white (and possibly correlated) corrections must be made to account for the change in spectrum and response properties of the isolated cells [26], [49], [50] (See Methods).

We note that Eq. (11), which is the basis of our iterative approach, provides an approximation to the network's output which is of higher than first order in connection strength. This may seem at odds with a theory that provides a linear correction to a cell's response, *cf.* Eq. (4). However, Eq. (11) does not capture nonlinear corrections to the response of individual cells, as the output of each cell is determined linearly from its input. It is the input that can contain terms of any order in connection strength stemming from directed paths of different lengths through the network.

We use the theoretical framework developed above to analyze the statistical structure of the spiking activity in a network of IF neurons described by Eq. (1). We first show that the cross-correlation functions between cells in two small networks can be studied in terms of contributions from directed paths through the network. We use a similar approach to understand the structure of correlations in larger all–to–all and random networks. We show that in networks where inhibition and excitation are tuned to exactly balance, only local interactions contribute to correlations. When such balance is broken by a relative elevation of inhibition, the result may be increased synchrony in the network. The theory also allows us to obtain averages of cross-correlation functions conditioned on connectivity between pairs of cells in random networks. Such averages can provide a tractable yet accurate description of the joint statistics of spiking in these networks.

The correlation structure is determined by the response properties of cells together with synaptic dynamics and network architecture. Network interactions are described by the matrix of synaptic filters, 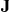, given in Eq. (2), while the response of cell 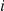 to an input is approximated using its linear response kernel 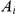. Synaptic dynamics, architecture, and cell responses are all combined in the matrix 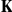, where 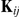 describes the response of cell 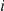 to an input from cell 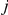 (See Eq. (1)). The correlation structure of network activity is approximated in Eq. (15) using the Fourier transforms of the interaction matrix, 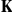, and the matrix of unperturbed autocorrelations 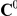.

### Statistics of the response of microcircuits

**Figure 3:**
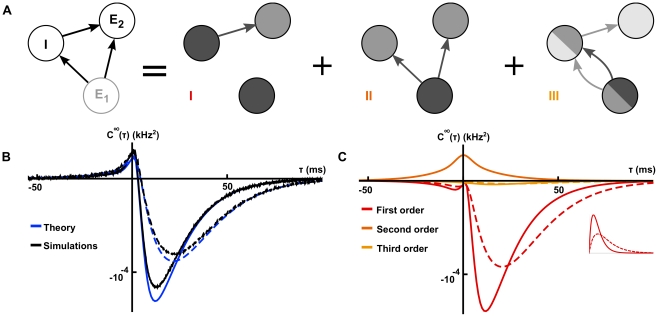
The relation between correlation structure and response statistics in a feed-forward inhibitory microcircuit. (**A**) The FFI circuit (left) can be decomposed into three submotifs. Equation (18) shows that each submotif provides a specific contribution to the cross-correlation between cells 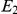 and 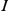. (**B**) Comparison of the theoretical prediction with the numerically computed cross-correlation between cells 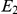 and 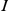. Results are shown for two different values of the inhibitory time constant, 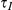 (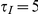 ms, solid line, 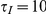 ms, dashed line). (**C**) The contributions of the different submotifs in panel A are shown for both 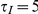 ms (solid) and 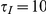 ms (dashed). Inset shows the corresponding change in the inhibitory synaptic filter. The present color scheme is used in subsequent figures. Connection strengths were 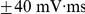 for excitatory and inhibitory connections. In each case, the long window correlation coefficient 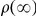 between the two cells was 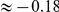.

We first consider a pair of simple microcircuits to highlight some of the features of the theory. We start with the three cell model of feed-forward inhibition (FFI) shown in Figure 3A [51]. The interaction matrix, 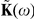, has the form
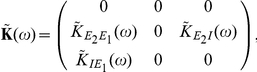
where cells are indexed in the order 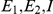. To simplify notation, we omit the dependence of 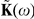 and other spectral quantities on 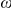.

Note that 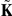 is nilpotent of degree 3 (that is, 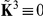), and the inverse of 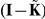 may be expressed as
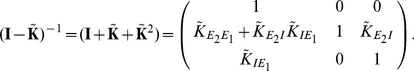
(17)Substituting Eq. (17) into Eq. (15) (and noting that a similar equation as Eq. (17) holds for 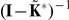) yields an approximation to the matrix of cross-spectra. For instance,
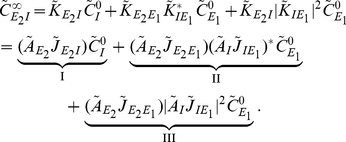
(18)
Figure 3B shows that these approximations closely match numerically obtained cross-correlations. 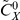 is the uncoupled power spectrum for cell 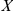.

Equation (18) gives insight into how the joint response of cells in this circuit is shaped by the features of the network. The three terms in Eq. (18) are directly related to the architecture of the microcircuit: Term I represents the correlating effect of the direct input to cell 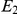 from cell 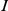. Term II captures the effect of the common input from cell 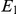. Finally, term III represents the interaction of the indirect input from 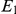 to 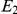 through 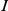 with the input from 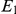 to 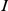 (See Figure 3C). A change in any single parameter may affect multiple terms. However, the individual contributions of all three terms are apparent.

To illustrate the impact of synaptic properties on the cross-correlation between cells 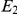 and 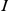 we varied the inhibitory time constant, 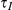 (See Figure 3B and C). Such a change is primarily reflected in the shape of the first order term, I: Multiplication by 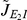 is equivalent to convolution with the inhibitory synaptic filter, 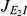. The shape of this filter is determined by 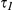 (See Eq. (2)), and a shorter time constant leads to a tighter timing dependency between the spikes of the two cells [24], [52]–[55]. In particular, Ostojic et al. made similar observations using a related approximation. In the FFI circuit, the first and second order terms, I and II, are dominant (red and dark orange, Figure 3B). The relative magnitude of the third order term, III (light orange, Figure 3B), is small. The next example shows that even in a simple recurrent circuit, terms of order higher than two may be significant.

**Figure 4:**
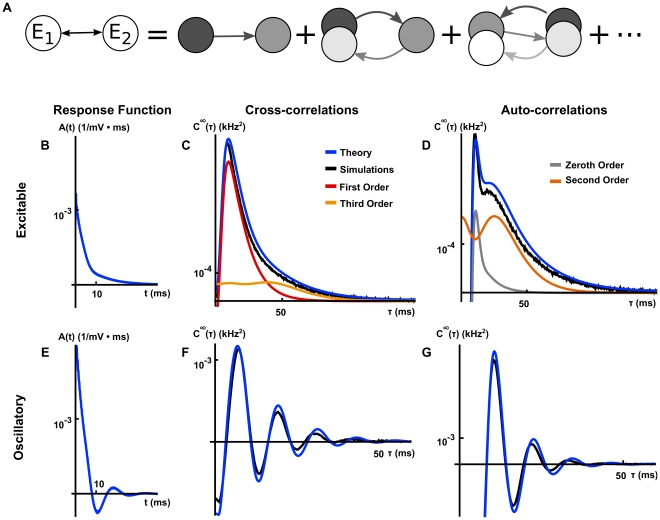
The relation between correlation structure and response statistics for two bidirectionally coupled, excitatory cells. (**A**) The cross-correlation between the two cells can be represented in terms of contributions from an infinite sequence of submotifs (See Eq. (20)). Though we show only a few “chain” motifs in one direction, one should note that there will also be contributions to the cross-correlation from chain motifs in the reverse direction in addition to indirect common input motifs (See the discussion of Figure 5). (**B**), (**E**) Linear response kernels in the excitable (B) and oscillatory (E) regimes. (**C**), (**F**) The cross-correlation function computed from simulations and theoretical predictions with first and third order contributions computed using Eq. (19) in the excitable (C) and oscillatory (F) regimes. (**D**), (**G**) The auto-correlation function computed from simulations and theoretical predictions with zeroth and second order contributions computed using Eq. (19) in the excitable (D) and oscillatory (G) regimes. In the oscillatory regime, higher order contributions were small relative to first order contributions and are therefore not shown. The network's symmetry implies that cross-correlations are symmetric, and we only show them for positive times. Connection strengths were 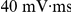. The long window correlation coefficient 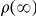 between the two cells was 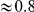 in the excitable regime and 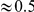 in the oscillatory regime. The ISI CV was approximately 0.98 for neurons in the excitable regime and 0.31 for neurons in the oscillatory regime.

More generally, the interaction matrices, 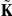, of recurrent networks are not nilpotent. Consider two reciprocally coupled excitatory cells, 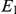 and 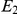 (See Figure 4A, left). In this case,
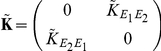
so that
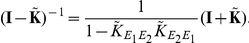
Equation (15) gives the following approximation to the matrix of cross-spectra
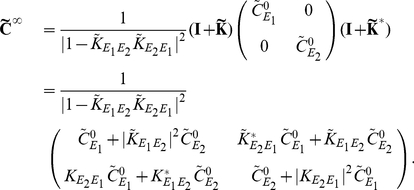
(19)In contrast to the previous example, this approximation does not terminate at finite order in interaction strength. After expanding, the cross-spectrum between cells 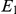 and 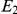 is approximated by
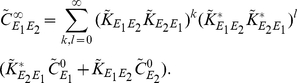
(20)Directed paths beginning at 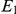 and ending at 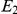 (or vice-versa) are of odd length. Hence, this approximation contains only odd powers of the kernels 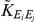, each corresponding to a directed path from one cell to the other. Likewise, the approximate power spectra contain only even powers of the kernels corresponding to directed paths that connect a cell to itself (See Figure 4A).

The contributions of different sub-motifs to the cross- and auto-correlations are shown in Figures 4C, D when the isolated cells are in a near-threshold excitable state (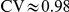). The auto-correlations are significantly affected by network interactions. We also note that chains of length two and three (the second and third submotifs in Figure 4A) provide significant contributions. Earlier approximations do not capture such corrections [24].

The operating point of a cell is set by its parameters (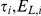, etc.) and the statistics of its input (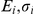). A change in operating point can significantly change a cell's response to an input. Using linear response theory, these changes are reflected in the response functions 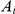, and the power spectra of the isolated cells, 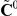. To highlight the role that the operating point plays in the approximation of the correlation structure given by Eq. (15), we elevated the mean and decreased the variance of background noise by increasing 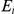 and decreasing 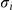 in Eq. (1). With the chosen parameters the isolated cells are in a super-threshold, low noise regime and fire nearly periodically (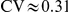). After the cells are coupled, this oscillatory behavior is reflected in the cross- and auto-correlations where the dominant contributions are due to first and zeroth order terms, respectively (See Figures 4F,G).

#### Orders of coupling interactions

**Figure 5:**
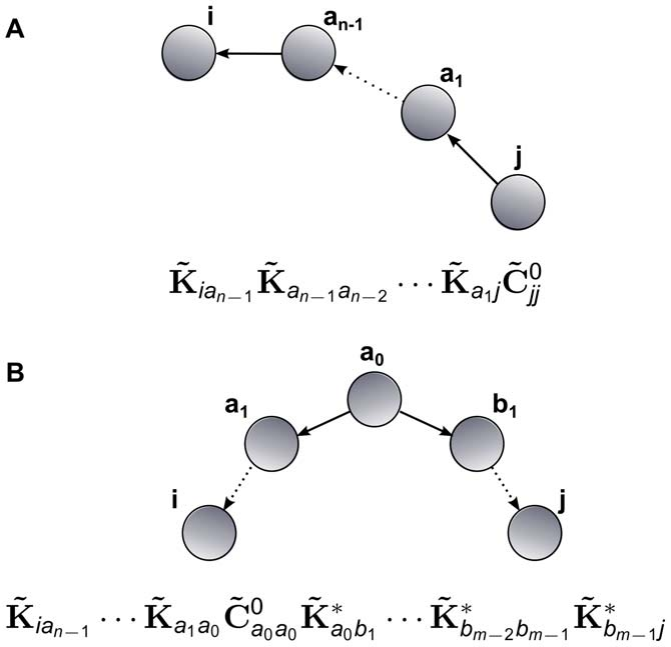
The motifs giving rise to terms in the expansion of **Eq. (15)**. (**A**) Terms containing only unconjugated (or only conjugated) interaction kernels 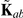 correspond to directed chains. (**B**) Terms containing both unconjugated and conjugated interaction kernels 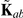 correspond to direct or indirect common input motifs.

**Figure 6:**
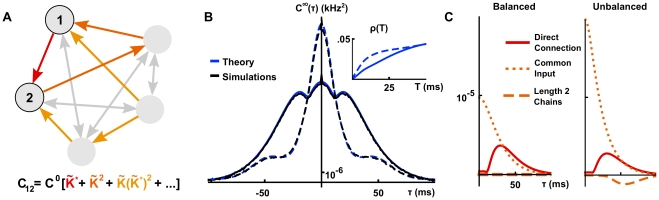
All–to–all networks and the importance of higher order motifs. (**A**) Some of the submotifs contributing to correlations in the all–to–all network. (**B**) Cross-correlations between two excitatory cells in an all–to-all network (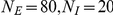) obtained using Eq. (21) (Solid – precisely tuned network with 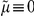 [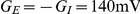 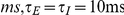], dashed – non-precisely tuned network with 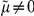 [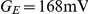 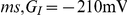 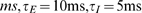]). (**C**) Comparison of first and second order contributions to the cross-correlation function in panel A in the precisely tuned (left) and non-precisely tuned (right) network. In both cases, the long window correlation coefficient 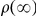 was 0.05.

It is often useful to expand Eq. (15) in terms of powers of 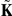 [31]. The term 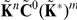 in the expansion is said to be of *order* 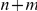. Equivalently, in the expansion of 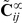, the order of a term refers to the sum of the powers of all constituent interaction kernels 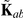. We can also associate a particular connectivity submotif with each term. In particular, 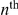 order terms of the form
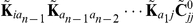
are associated with a directed path 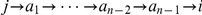 from cell 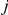 to cell 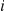. Similarly, the term 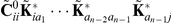 corresponds to a 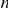-step path from cell 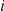 to cell 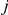. An 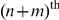 order term of the form
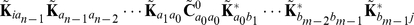
represents the effects of an indirect common input 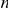 steps removed from cell 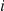 and 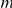 steps removed from cell 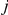. This corresponds to a submotif of the form 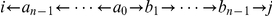 consisting of two branches originating at cell 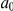. (See Figure 5, and also Figure 6A and the discussion around Eqs. (18,20).)

### Statistics of the response of large networks

The full power of the present approach becomes evident when analyzing the activity of larger networks. We again illustrate the theory using several examples. In networks where inhibition and excitation are tuned to be precisely balanced, the theory shows that only local interactions contribute to correlations. When this balance is broken, terms corresponding to longer paths through the network shape the cross-correlation functions. One consequence is that a relative increase in inhibition can lead to elevated network synchrony. We also show how to obtain tractable and accurate approximation of the average correlation structure in random networks.

#### A symmetric, all–to–all network of excitatory and inhibitory neurons

We begin with an all–to–all coupled network of 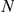 identical cells. Of these cells, 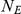 make excitatory, and 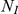 make inhibitory synaptic connections. The excitatory cells are assigned indices 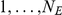, and the inhibitory cells indices 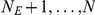. All excitatory (inhibitory) synapses have weight 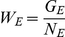 (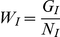), and timescale 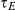 (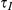). The interaction matrix 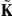 may then be written in block form,
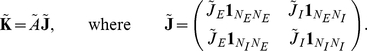
Here 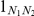 is the 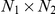 matrix of ones, 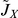 is the weighted synaptic kernel for cells of class 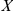 (assumed identical within each class), and 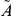 is the susceptibility function for each cell in the network. Although the effect of autaptic connections (those from a cell to itself) is negligible (See Figure S2 in Text S1), their inclusion significantly simplifies the resulting expressions.

We define 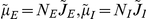, and 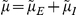. Using induction, we can show that
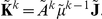
Direct matrix multiplication yields
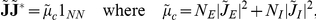
which allows us to calculate the powers 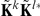 when 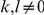,
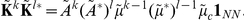
An application of Eq. (15) then gives an approximation to the matrix of cross-spectra:
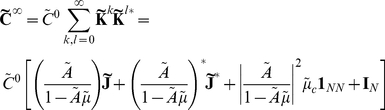
(21)The cross-spectrum between two cells in the network is therefore given by
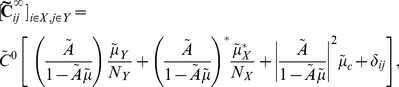
(22)where 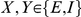. In Eq. (22) the first two terms represent the effects of all unidirectional chains originating at cell 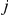 and terminating at cell 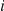, and vice versa. To see this, one should expand the denominators as power series in 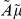. The third term represents the effects of direct and indirect common inputs to the two neurons, which can be seen by expanding this denominator as a product of power series in 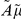 and 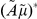. In Figure 6A, we highlight a few of these contributing motifs.

Interestingly, when excitation and inhibition are tuned for precise balance (so that the mean excitatory and inhibitory synaptic currents cancel, and 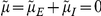). Using 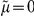 in Eq. (22) yields
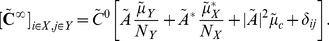
(23)Effects of direct connections between the cells are captured by the first two terms, while those of direct common inputs to the pair are captured by the third term. Contributions from other paths do not appear at any order. In other words, *in the precisely balanced case only local interactions contribute to correlations.*

To understand this cancelation intuitively, consider the contribution of directed chains originating at a given excitatory neuron, 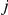. For 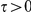, the cross-correlation function, 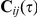, is determined by the change in firing rate of cell 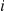 at time 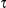 given a spike in cell 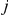 at time 0. By the symmetry of the all–to–all connectivity and stationarity, the firing of cell 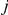 has an equal probability of eliciting a spike in any excitatory or inhibitory cell in the network. Due to the precise synaptic balance, the postsynaptic current generated by the elicited spikes in the excitatory population will cancel the postsynaptic current due to elicited spikes in the inhibitory population on average. The contribution of other motifs cancel in a similar way.

In Figure 6B, we show the impact of breaking this excitatory-inhibitory balance on cross-correlation functions. We increased the strength and speed of the inhibitory synapses relative to excitatory synapses, while holding constant, for sake of comparison, the long window correlation coefficients 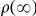 between excitatory pairs (note that, by symmetry, all excitatory pairs should have the same correlation coefficient). Moreover, the degree of network synchrony, characterized by the short window correlation coefficients, is increased (See Figure 6B inset). Intuitively, a spike in one of the excitatory cells transiently increases the likelihood of spiking in all other cells in the network. Since inhibition in the network is stronger and faster than excitation, these additional spikes will transiently decrease the likelihood of spiking in twice removed cells.

Linear response theory allows us to confirm this heuristic observation, and quantify the impact of the imbalance on second order statistics. Expanding Eq. (22) for two excitatory cells to second order in coupling strength, we find
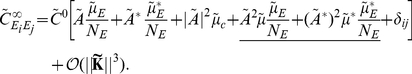
(24)Compared to the balanced case, there is no longer a complete cancellation between contributions of chains involving excitatory and inhibitory cells, and the two underlined terms appear as a result (compare with Eq. (23)). These terms capture the effects of all length two chains between cells 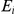 or 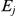, starting at one and terminating at the other. The relative strengthening of inhibition implies that chains of length two provide a negative contribution to the cross-correlation function at short times (*cf.* [56], see the dashed orange lines in Figure 6C). Additionally, the impact of direct common input to cells 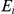 and 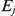 on correlations is both larger in magnitude (because we increased the strength of both connection types) and sharper (the faster inhibitory time constant means common inhibitory inputs induce sharper correlations). These changes are reflected in the shape of the second order, common input term 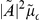 in Eq. (24) (see dotted orange lines in Figure 6C).

In sum, unbalancing excitatory and inhibitory connections via stronger, faster inhibitory synapses enhances synchrony, moving a greater proportion of the covariance mass closer to 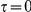 (See Figure 6B). To illustrate this effect in terms of underlying connectivity motifs, we show the contributions of length two chains and common input in both the precisely tuned and non-precisely tuned cases in Figure 6C. A similar approach would allow us to understand the impact of a wide range of changes in cellular or synaptic dynamics on the structure of correlations across networks.

#### Random, fixed in-degree networks of homogeneous excitatory and inhibitory neurons

Connectivity in cortical neuronal networks is typically sparse, and connection probabilities can follow distinct rules depending on area and layer [57]. The present theory allows us to consider arbitrary architectures, as we now illustrate.

We consider a randomly connected network of 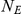 excitatory and 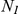 inhibitory cells coupled with probability 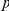. To simplify the analysis, every cell receives exactly 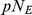 excitatory and 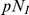 inhibitory inputs. Thus, having fixed in-degree (that is, the number of inputs is fixed and constant across cells), each cell receives an identical level of mean synaptic input. In addition, we continue to assume that cells are identical. Therefore, the response of each cell in the network is described by the same linear response kernel. The excitatory and inhibitory connection strengths are 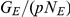 and 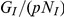, respectively. The timescales of excitation and inhibition may differ, but are again identical for cells within each class.

**Figure 7:**
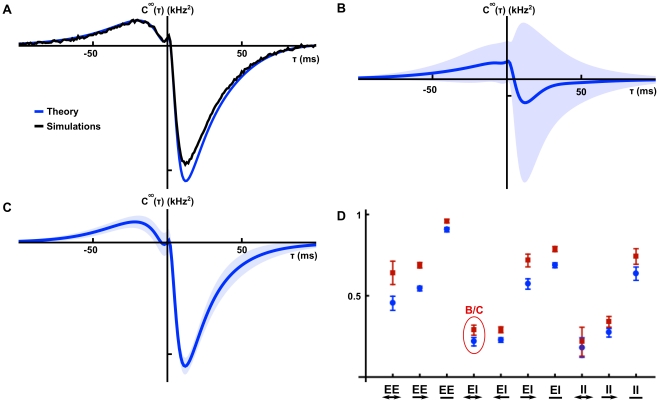
Correlations in random, fixed in-degree networks. (**A**) A comparison of numerically obtained excitatory-inhibitory cross-correlations to the approximation given by Eq. (26). (**B**) Mean and standard deviation for the distribution of correlation functions for excitatory-inhibitory pairs of cells. (Solid line – mean cross-correlation, shaded area – one standard deviation from the mean, calculated using bootstrapping in a single network realization). (**C**) Mean and standard deviation for the distribution of cross-correlation functions conditioned on cell type *and* first order connectivity for a reciprocally coupled excitatory-inhibitory pair of cells. (Solid line – mean cross-correlation function, shaded area – one standard deviation from the mean found by bootstrapping). (**D**) Average reduction in 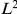 error between cross-correlation functions and their respective first-order conditioned averages, relative to the error between the cross-correlations and their cell-type averages. Blue circles give results for a precisely tuned network, and red squares for a network with stronger, faster inhibition. Error bars indicate two standard errors above and below the mean. 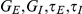 for panels A-C are as in the precisely tuned network of Figure 6, and the two networks of panel D are as in the networks of the same figure.

The approximation of network correlations (Eq. (15)) depends on the realization of the connectivity matrix. For a fixed realization, the underlying equations can be solved numerically to approximate the correlation structure (See Figure 7A). However, the cross-correlation between a pair of cells of given types has a form which is easy to analyze when only leading order terms in 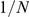 are retained.

Specifically, the average cross-spectrum for two cells of given types is (See Section 1 in Text S1)
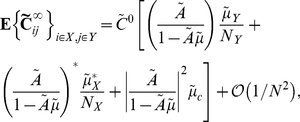
(25)when 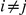. This shows that, to leading order in 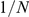, the mean cross-spectrum between two cells in given classes equals that in the all–to–all network (see Eq. (22)). Therefore our previous discussion relating network architecture to the shape of cross-correlations in the all–to–all network extends to the average correlation structure in the random network for large 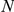.

Pernice et al. [31] derived similar expressions for the correlation functions in networks of interacting *Hawkes processes* [58], [59], which are linear, self-exciting point processes with history-dependent intensities. They assumed that either the network is regular (i.e., both in- and out-degrees are fixed) or has a sufficiently narrow degree distribution. Our analysis depends on having fixed in-degrees, and we do not assume that networks are fully regular. Both approaches lead to results that hold approximately (for large enough 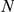) when the in-degree is not fixed.

#### Average correlations between cells in the random network conditioned on first order connectivity

As Figure 7B shows there is large variability around the mean excitatory-inhibitory cross-correlation function given by the leading order term of Eq. (25). Therefore, understanding the average cross-correlation between cells of given types does not necessarily provide much insight into the mechanisms that shape correlations on the level of individual cell pairs. Instead, we examine the average correlation between a pair of cells conditioned on their first order (direct) connectivity.

We derive expressions for first order conditional averages correct to 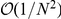 (See Section 2 in Text S1). The average cross-spectrum for a pair of cells with indices 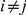, conditioned on the value of the direct connections between them is
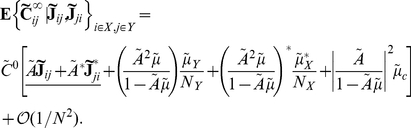
(26)Here we set 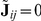 if we condition on the absence of a connection 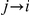, and 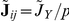 if we condition on its presence. The term 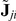 is set similarly.

Although Eq. (26) appears significantly more complicated than the cell-type averages given in Eq. (25), they only differ in the underlined, first order terms. The magnitude of expected contributions from all higher order motifs is unchanged and coincides with those in the all–to–all network.

Figure 7C shows the mean cross-correlation function for mutually coupled excitatory-inhibitory pairs. Taking into account the mutual coupling significantly reduces variability (Compare with Figure 7B). To quantify this reduction, we calculate the mean reduction in variability when correlation functions are computed conditioned on the connectivity between the cells. For a single network, the relative decrease in variability can be quantified using
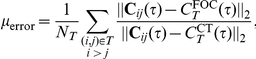
where 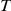 represents pairs of cells of a given type and connection (in the present example these are reciprocally coupled excitatory-inhibitory pairs), 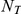 is the number of pairs of that type in the network, 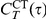 is the leading order approximation of average correlations given only the type of cells in 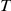 (as in Eq. (25)), and 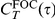 the leading order approximation to average correlations conditioned on the first order connectivity of class 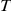 (as in Eq. (26)). We make use of the norm 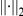 defined by 
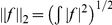
. Figure 7D shows 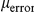 averaged over twenty networks. In particular, compare the reduction in variability when conditioning on bidirectional coupling between excitatory-inhibitory pairs shown in Figures 7B,C, with the corresponding relative error in Figure 7D (circled in red).

## Discussion

We have extended and further developed a general theoretical framework that can be used to describe the correlation structure in a network of spiking cells. The application of linear response theory allows us to find tractable approximations of cross-correlation functions in terms of the network architecture and single cell response properties. The approach was originally used to derive analytical approximations to auto- and cross-spectra in an all–to–all inhibitory network in order to study the population response of the electrosensory lateral line lobe of weakly electric fish [26]. The key approximation relies on the assumption that the activity of cells in the network can be represented by a mixed point and continuous stochastic process, as given in Eq. (9). This approximation may be viewed as a generalization of classic Linear-Poisson models of neural spiking: the crucial difference is the replacement of the stationary firing rate by a realization of an integrate-and-fire spiking process. This allows for the retention of the underlying IF spiking activity while additionally posing that neurons act as perfect linear filters of their inputs. An iterative construction then leads to the expressions for approximate cross-correlations between pairs of cells given by Eq. (15).

The linear response framework of Lindner et al. [26] was extended by Marinazzo et al. [60] to somewhat more complex networks, and compared with other studies in which networks exhibit collective oscillations. In addition, other works [13], [61], [62] used linear response techniques to study information in the collective response of cells in a network. More recently, Ostojic et al. [24] obtained formulas for cross-correlations given in Eq. (9), which correspond to the first step in the iterative construction. Their approach captures corrections due to direct coupling (first order terms) and direct common input (second order terms involving second powers of interaction kernels; see also [49], [63]). Our approach can be viewed as a generalization that also accounts for length two directed chains, along with all higher order corrections. As Figure 4 illustrates, these additional terms can be significant. The present approach also allows us to calculate corrected auto-correlations, in contrast with that of Ostojic et al.

Our work is also closely related to that of Pernice et al. [31], who analyzed the correlation structure in networks of interacting *Hawkes processes* [58], [59]. Both studies represent correlations between cell pairs in terms of contributions of different connectivity motifs. However, our methods also differ: while their expressions are exact for Hawkes processes, Pernice et al. did not compare their results to those obtained using physiological models, and did not account for the response properties of individual cells (though it is possible that both can be achieved approximately by using appropriate kernels for the Hawkes processes). Moreover, for simplicity Pernice et al. examined only “total” spike count covariances, which are the integrals of the cross-correlation functions. However, as they note, their approach can be extended to obtain the temporal structure of cross-correlations. Similarly, Toyoizumi et al. [64] derive expressions for cross-correlations in networks of interacting point process models in the Generalized Linear Model (GLM) class. These are very similar to Hawkes processes, but feature a static nonlinearity that shapes the spike emission rate.

To illustrate the power of the present linear response theory in analyzing the factors that shape correlations, we considered a number of simple examples for which the approximation given by Eq. (15) is tractable. We showed how the theory can be used both to gain intuition about the network and cell properties that shape correlations, and to quantify their impact. In particular, we explained how only local connections affect correlations in a precisely tuned all–to–all network, and how strengthening inhibition may synchronize spiking activity. In each case, we use comparisons with integrate-and-fire simulations to show that linear response theory makes highly accurate predictions.

It may be surprising that *linear* response theory can be used to provide corrections to cross-correlations of arbitrary order in network connectivity. The key to why this works lies in the accuracy of the linearization. A more accurate approximation could be obtained by including second and higher order corrections to the approximate response of a single cell, as well as corrections to the joint response. While including such terms is formally necessary to capture all contributions of a given order in network connectivity [32], [33], the success of of linear response theory suggests that they are small for the cases at hand. In short, the present approximation neglects higher-order corrections to the approximate response of individual cells, along with all corrections involving joint responses, but accounts for paths through the network of arbitrary length.

As expected from the preceding discussion, simulations suggest that, for IF neurons, our approximations become less accurate as cells receive progressively stronger inputs. The physical reasons for this loss of accuracy could be related to interactions between the “hard threshold” and incoming synaptic inputs with short timescales. Additionally, while the theory will work for short synaptic timescales, it will improve for slower synaptic dynamics, limiting towards being essentially exact in the limit of arbitrarily long synaptic time constants (note the improvement in the approximation for the FFI circuit for the slower timescale exhibited in Figure 3). Another important factor is background noise, which is known to improve the accuracy of the linear description of single cell responses. We assume the presence of a white noise background, although it is possible to extend the present methods to colored background noise [25], [65].

We found that linear response theory remains applicable in a wide range of dynamical regimes, including relatively low noise, superthreshold regimes where cells exhibit strong oscillatory behavior. Moreover, the theory can yield accurate approximations of strong correlations due to coupling: for the bidirectionally coupled excitatory circuit of Figure 4, the approximate cross-correlations match numerically obtained results even when correlation coefficients are large (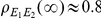 in the excitable regime, 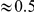 in the oscillatory regime). Additional discussion of the limits of applicability of linear response to the computation of correlations in networks can be found in the Supplementary Information. There, we show that the approximation is valid over a range of physiological values in the case of the all-to-all network, and that the theory gives accurate predictions in the presence of low firing rates (see Figures S3, S4 in Text S1).

The limits of linear response approximations of time-dependent firing activity and correlations have been tested in a number of other studies. Ostojic and Brunel [66] examined this accuracy in the relatively simple case of a neuron receiving filtered Gaussian noise in addition to a white background. Chacron et al. [61] noted that linear response approaches applied to networks of perfect integrators begin to display significant errors at larger connection strengths. Marinazzo et al. [60] remarked on the errors induced by network effects in linear response approximations to correlations in a delayed feedback loop. In particular, these errors were attributed to network effects such as synchrony in the excitatory population. The authors noted that such activity can not be correctly modeled by a linear approach.

Although we have demonstrated the theory using networks of integrate–and–fire neurons, the approach is widely applicable. The linear response kernel and power spectrum for a general integrate and fire neuron model can be easily obtained [29]. In addition, it is also possible to obtain the rate, spectrum, and susceptibility for modulation of the mean conductance in the case of conductance-based (rather than current-based) synapses (See [67] and Section 3 in Text S1). As the linear response kernel is directly related to the spike triggered average [24], [30], the proposed theoretical framework should be applicable even to actual neurons whose responses are characterized experimentally.

The possibilities for future applications are numerous. For example, one open question is how well the theory can predict correlations in the presence of adaptive currents [67]. In addition, the description of correlations in terms of architecture and response properties suggests the possibility of addressing the difficult inverse problem of inferring architectural properties from correlations [22]–[24], [64]. Ostojic et al. applied linear response methods to the latter problem. It is our hope that the present approach will prove a valuable tool in moving the computational neuroscience community towards a more complete understanding of the origin and impact of correlated activity in neuronal populations.

## Methods

### Measures of spike time correlation

We quantify dependencies between the responses of cells in the network using the spike train auto- and cross-correlation functions [39]. For a pair of spike trains, 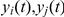, the cross-correlation function 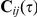 is defined as
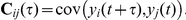
The auto-correlation function 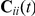 is the cross-correlation between a spike train and itself, and 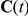 is the matrix of cross-correlation functions. Denoting by 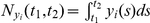 the number of spikes over a time window 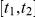, the spike count correlation, 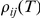, over windows of length 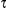 is defined as,
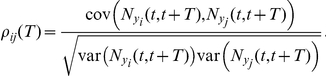
We assume stationarity of the spiking processes (that is, the network has reached a steady state) so that 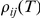 does not depend on 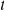. We also use the total correlation coefficient 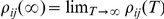 to characterize dependencies between the processes 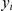 and 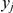 over arbitrarily long timescales.

The spike count covariance is related to the cross-correlation function by [7], [68]

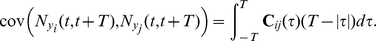
We can interpret the cross-correlation as the conditional probability that cell 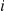 spikes at time 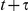 given that cell 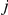 spiked at time 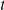. The conditional firing rate,
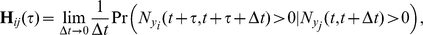
is the firing rate of cell 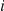 conditioned on a spike in cell 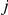 at 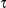 units of time in the past, and 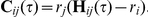


Define the Fourier transform of a function 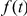 as 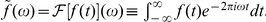 We will often make use of the cross-spectrum between the output of cells 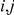, given by 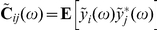, which is the Fourier transform of the cross-correlation function of cells 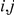. The power spectrum 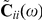 is the cross-spectrum between a cell and itself, and is the Fourier transform of the auto-correlation function.

#### Numerical methods

Simulations were run in C++, and the stochastic differential equations were integrated with a standard Euler method with a time-step of 0.01 ms. General parameter values were as follows: 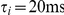, 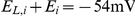, 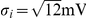, 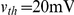, 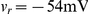, 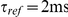, 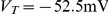, 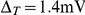, 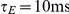, 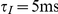, 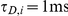. Marginal statistics (firing rates, uncoupled power spectra and response functions) were obtained using the threshold integration method of [29] in MATLAB. We have posted a package of code which contains examples of all the numerical methods used in this paper (both simulations and theory) at http://www.math.uh.edu/~josic/myweb/software.html. Additional code is available upon request.

#### Calculation of stationary rates in a recurrent network

The stationary firing rate of an IF neuron can be computed as a function of the mean and intensity of internal noise (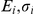) and other cellular parameters (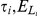, etc…) [69]. Denote the stationary firing rate of cell 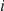 in the network by 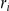, and by 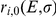 the stationary firing rate in the presence of white noise with mean 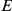 and variance 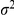. We keep the dependencies on other parameters are implicit. The stationary rates, 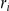, in the recurrent network without external input are determined self-consistently by
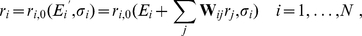
where we used 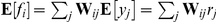. This equality holds because the synaptic kernels, 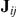, were normalized to have area 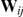. These equations can typically be solved by fixed-point iteration.

Note that this provides an effective mean input, 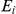, to each cell, but does not give adjustments to the variance, 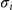. We assume that the major impact of recurrent input is reflected in 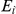, and ignore corrections to the cell response involving higher order statistics of the input. This approach is valid as long as fluctuations in the recurrent input to each cell are small compared to 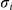, and may break down otherwise [27].

#### Correction to statistics in the presence of an external white noise signals

Expression (16) can be used to compute the statistics of the network response to inputs 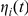 of finite variance. As noted by [26], when inputs have infinite variance additional corrections are necessary. As a particular example, consider the case where the processes are correlated white noise, i.e., when 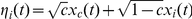, where 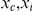 are independent white noise processes with variance 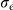. Then each 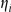 is also a white noise process with intensity 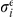, but 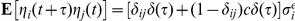. The firing rate of cell 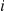 in response to this input is 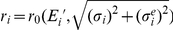, and the point around which the response of the cell is linearized needs to be adjusted.

Finally, we may apply an additional correction to the linear response approximation of autocorrelations. For simplicity, we ignore coupling in Eq. (16) (so that 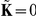). Linear response predicts that 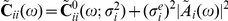, where we have introduced explicit dependence on 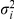, the variance of white noise being received by an IF neuron with power spectrum 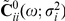, in the absence of the external signal. The approximation may be improved in this case by making the following substitution in Eq. (16) [26], [50]:
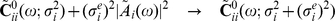
The response function 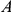 should be adjusted likewise.

#### Convolution of matrices

Let 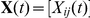 and 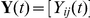 be 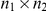 and 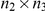 matrices of functions, respectively. We define the convolution of matrices 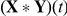 to be the 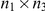 matrix of functions with entries defined by
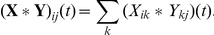
Expectations and convolutions commute for matrix convolutions as matrix expectations are taken entry-wise. Each entry of a matrix convolution is a linear combination of scalar convolutions which commute with expectations. Additionally, we adopt the convention that the zeroth power of the interaction matrix, 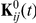, is the diagonal matrix with 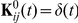 when 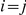. Hence 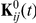 acts as the identity matrix under matrix convolution.

## Supporting Information

Text S1 Supplementary information file containing derivations and additional content, such as an exploration of the error of the theory. Supporting information figures were included in this file (and not separately).
(PDF)
