# Supplementary material for: Impact of Network Structure and Cellular Response on Spike Time Correlations
Source: PLoS Comput Biol. 2012 Mar 22;8(3):e1002408. doi: 10.1371/journal.pcbi.1002408 (PMC3310711; doi:10.1371/journal.pcbi.1002408)
Supplement: Text S1 — Supplementary information file containing derivations and additional content, such as an exploration of the error of the theory. Supporting information figures were included in this file (and not separately). (PDF) [file pcbi.1002408.s001.pdf]

Impact of network structure and cellular response on spike time correlations - Text S1 (Supporting Information)

James Trousdale[1,*], Yu Hu[2], Eric Shea-Brown[2], Krešimir Josić[1,3]
1 Department of Mathematics, University of Houston, Houston, TX 77204, USA
2 Department of Applied Mathematics, Program in Neurobiology and Behavior, University of Washington, Seattle, WA 98195, USA
3 Department of Biology and Biochemistry, University of Houston, Houston, TX 77204, USA
∗ E-mail: jrtrousd@math.uh.edu

1 Cell-type average correlations in random networks

1.1 Setup

Consider a network consisting of N cells, of which $N_E = f_E N$ are excitatory, and $N_I = f_I N$ inhibitory. Synaptic connections between the cells are random, and the probability of a contact is p. Additionally, the number of excitatory and inhibitory inputs to each cell (henceforth the in-degrees) is fixed to pN_E and pN_I respectively. The strength of an excitatory connection, defined by the total area under a post-synaptic potential, is $G_E/(pN_E)$, and the strength of an inhibitory connection is $G_I/(pN_I)$. Excitatory and inhibitory inputs follow the time course described by the normalized (unit-area) synaptic kernels with frequency domain representation $\tilde{F}_E$ and $\tilde{F}_I$, respectively.

Under these conditions, the interaction matrix $\tilde{\mathbf{K}}$ takes the form

$$\tilde{\mathbf{K}}(\omega) = \tilde{A}(\omega)\tilde{\mathbf{J}}(\omega) \quad \text{where} \quad \tilde{\mathbf{J}}(\omega) = \begin{pmatrix} \frac{G_E}{pN_E}\tilde{F}_E(\omega)\mathbf{R}_{N_E N_E} & \frac{G_I}{pN_I}\tilde{F}_I(\omega)\mathbf{R}_{N_E N_I} \\ \frac{G_E}{pN_E}\tilde{F}_E(\omega)\mathbf{R}_{N_I N_E} & \frac{G_I}{pN_I}\tilde{F}_I(\omega)\mathbf{R}_{N_I N_I} \end{pmatrix}, \tag{1}$$

where, $\mathbf{R}_{N_1 N_2}$ is an $N_1 \times N_2$ matrix of 0's and 1's, with pN_2 randomly chosen non-zero entries on each row. Note that entries of $\tilde{\mathbf{K}}(\omega)$ are independent except within the rows of each block. Moving forward, we will suppress dependencies on ω for notation. The derived expressions can be assumed to hold for each ω.

In the following, $\mathbf{E}\{\cdot\}$ is an average over realizations of the random adjacency matrices $\mathbf{R}_{XY}$. We define $\tilde{\mu}_X$ to be

$$\begin{aligned} \tilde{\mu}_X = \sum_{k_0 \in X} \mathbf{E}\Big\{\tilde{\mathbf{J}}_{k_1 k_0}\Big\} &= N_X \mathbf{E}\Big\{\tilde{\mathbf{J}}_{k_1 k_0}\Big\}_{k_0 \in X} \\ &= N_X \left(\frac{G_X}{pN_X}\tilde{F}_E\right) p \\ &= G_X \tilde{F}_X \end{aligned} \tag{2}$$

where we have used the subscript notation $k_0 \in X$ to denote that the cell with index k_0 belongs to class $X = E$ or $X = I$. Note that the definition of $\tilde{\mu}_X$ is independent of the index k_1 chosen, and may alternatively be defined as the same value via a simple sum without expectations as we assumed in-degrees to be fixed. We also define $\tilde{\mu}$ as

$$\tilde{\mu} = \sum_{k_0} \mathbf{E}\Big\{\tilde{\mathbf{J}}_{k_1 k_0}\Big\} = \tilde{\mu}_E + \tilde{\mu}_I = G_E\tilde{F}_E + G_I\tilde{F}_I.$$

and $\tilde{\mu}_c$ as

$$\begin{aligned}
\tilde{\mu}_c &= \sum_{k_0} \mathbf{E}\Big\{\tilde{\mathbf{J}}_{k_1k_0}\tilde{\mathbf{J}}^*_{l_1k_0}\Big\} \\
&= N_E\mathbf{E}\Big\{\tilde{\mathbf{J}}_{k_1k_0}\tilde{\mathbf{J}}^*_{l_1k_0}\Big\}_{k_0\in E} + N_I\mathbf{E}\Big\{\tilde{\mathbf{J}}_{k_1k_0}\tilde{\mathbf{J}}^*_{l_1k_0}\Big\}_{k_0\in I} \\
&= N_E\left|\frac{G_E}{pN_E}\tilde{F}_E\right|^2 p^2 + N_I\left|\frac{G_I}{pN_I}\tilde{F}_I\right|^2 p^2 \\
&= \frac{1}{N_E}|\tilde{\mu}_E|^2 + \frac{1}{N_I}|\tilde{\mu}_I|^2.
\end{aligned} \tag{3}$$

where the definition of $\tilde{\mu}_c$ is independent of the indices k_1, l_1, so long as $k_1 \neq l_1$. To understand this calculation, as we sum over k_0, we will sum over N_E excitatory cells and N_I inhibitory cells. For each value of k_0, conditioned on the type of cell being $k_0 \in X$, the squared absolute value of the matrix entry will be $|\frac{\tilde{\mu}_X}{pN_E}|^2$ with probability p^2 (the probability both connections are "on"), and zero otherwise.

1.2 Approximate independence of the entries of J

Consider a pair of cells with indices i, j in the random balanced network. The linear response approximation to the correlation between cells i and j in the present network is

$$\begin{aligned}
\mathbf{E}\Big\{\tilde{\mathbf{C}}_{ij}\Big\}_{i\in X, j\in Y} &= \mathbf{E}\Big\{[(I-\tilde{A}\tilde{\mathbf{J}})^{-1}(I-\tilde{A}^*\tilde{\mathbf{J}}^*)^{-1}\tilde{C}^0]_{ij}\Big\}_{i\in X, j\in Y} \\
&= \tilde{C}_0\sum_{n,m=0}^{\infty}\tilde{A}^n(\tilde{A}^*)^m\mathbf{E}\Big\{[\tilde{\mathbf{J}}^n(\tilde{\mathbf{J}}^*)^m]_{ij}\Big\}_{i\in X, j\in Y}.
\end{aligned} \tag{4}$$

We will assume that this series converges.

The assumption of fixed in-degrees means that there are dependencies between the entries of $\tilde{\mathbf{J}}$ inside each row of each block of the matrix which are absent in a fully random network. The matrix consists of four blocks, corresponding to the pair types of pre- and post-synaptic cells (EE, EI, IE and II). However, if the network is large and connections are relatively weak, then the entries of the matrix $\tilde{\mathbf{J}}$ are approximately independent. In particular, we will show that

$$\begin{aligned}
&\sum_{k_0,\ldots,k_{n-1},l_1,\ldots,l_{m-1}=1}^{N}\Big[\mathbf{E}\Big\{\tilde{\mathbf{J}}_{ik_{n-1}}\tilde{\mathbf{J}}_{k_{n-1}k_{n-2}}\cdots\tilde{\mathbf{J}}_{k_1k_0}\tilde{\mathbf{J}}^*_{l_1k_0}\cdots\tilde{\mathbf{J}}^*_{jl_{m-1}}\Big\} \\
&-\mathbf{E}\Big\{\tilde{\mathbf{J}}_{ik_{n-1}}\Big\}\mathbf{E}\Big\{\tilde{\mathbf{J}}_{k_{n-1}k_{n-2}}\Big\}\cdots\mathbf{E}\Big\{\tilde{\mathbf{J}}_{k_1k_0}\left(\tilde{\mathbf{J}}_{l_1k_0}\right)^*\Big\}\cdots\mathbf{E}\Big\{\left(\tilde{\mathbf{J}}_{jl_{m-1}}\right)^*\Big\}\Big] \sim \mathcal{O}(1/N^2)
\end{aligned} \tag{5}$$

where we have neglected the conditioning of the cell types of i, j for notation, though this conditioning may be assumed to always be present. This means that, to lowest order in $1/N$, expectations of powers of $\tilde{\mathbf{J}}$ may be taken as if all entries of $\tilde{\mathbf{J}}$ were chosen completely independently, and without concern for the fixing of in-degrees. Note that the term $\mathbf{E}\Big\{\tilde{\mathbf{J}}_{k_1k_0}\left(\tilde{\mathbf{J}}_{l_1k_0}\right)^*\Big\}$ is not factored - these two terms will not be approximately independent as they both involve a connection originating from the same cell k_0, meaning they will (when the connections are present) have the same value for any k_0. However, there is no need to assume their approximate independence, as when the sum of these terms is taken across k_0, the value is $\tilde{\mu}_c$ for any k_1, l_1.

1.2.1 Case 1: $n = 1, m = 0$

These values of n, m correspond to the correlating effects of direct synaptic interactions between pairs of cells. In this case, we are just looking at $\mathbf{E}\{\mathbf{J}_{ij}\}$. We have by definition

$$\mathbf{E}\Big\{\tilde{\mathbf{J}}_{ij}\Big\}_{i\in X, j\in Y} = \frac{\tilde{\mu}_Y}{N_Y}, \tag{6}$$

as the total amount of input from cells of class Y is $\tilde{\mu}_Y$, so that the expected impact of a single connection is as stated.

1.2.2 Case 2: $n = m = 1$

These values of n, m correspond to the correlating effects of direct shared inputs to pairs of cells in the network. In this case, we can find the exact value of $\mathbf{E}\Big\{[\tilde{\mathbf{J}}\tilde{\mathbf{J}}^*]_{ij}\Big\}$. First, suppose $i \neq j$, then directly from the definition of $\tilde{\mu}_c$, we have

$$\mathbf{E}\Big\{[\tilde{\mathbf{J}}\tilde{\mathbf{J}}^*]_{ij}\Big\}_{i\in X, j\in Y} = \sum_{k=1}^{N} \mathbf{E}\Big\{\tilde{\mathbf{J}}_{ik}\tilde{\mathbf{J}}^*_{jk}\Big\} = \tilde{\mu}_c \tag{7}$$

and if $i = j$, we find

$$\mathbf{E}\Big\{[\tilde{\mathbf{J}}\tilde{\mathbf{J}}^*]_{ii}\Big\}_{i\in X} = \sum_{k=1}^{N} \mathbf{E}\Big\{|\tilde{\mathbf{J}}_{ik}|^2\Big\} = N_E \left|\frac{G_E}{pN_E}\tilde{F}_E\right|^2 p + N_I \left|\frac{G_I}{pN_I}\tilde{F}_I\right|^2 p = \frac{\tilde{\mu}_c}{p} \tag{8}$$

1.2.3 Case 3: $2 \leq n \ll N, m = 0$

These values of n, m correspond to correlating effects of directed chains between cells in the network. Following Eq. (5) for terms of this form, we must examine sums of the form

$$\sum_{k_1,\ldots,k_{n-1}=1}^{N} \Big[\mathbf{E}\Big\{\tilde{\mathbf{J}}_{ik_{n-1}}\cdots\tilde{\mathbf{J}}_{k_2k_1}\tilde{\mathbf{J}}_{k_1j}\Big\} - \mathbf{E}\Big\{\tilde{\mathbf{J}}_{ik_{n-1}}\Big\}\cdots\mathbf{E}\Big\{\tilde{\mathbf{J}}_{k_2k_1}\Big\}\mathbf{E}\Big\{\tilde{\mathbf{J}}_{k_1j}\Big\}\Big]$$

There will be N^{n-1} terms in this sum and the number of terms which factor (and cancel) will be at least the number of ways to pick different arrangements of $n-1$ distinct integers from $1, \ldots, i-1, i+1, \ldots, N$, or $P(N-1, n-1)$. If $n \ll N$, then $P(N-1, n-1) = N^{n-1} + \mathcal{O}(N^{n-2})$, so that only $\mathcal{O}(N^{n-2})$ terms do not cancel. Since each term is proportional to $1/N^n$, the error introduced by assuming independence of matrix entries when averaging is $\mathcal{O}(1/N^2)$. Hence, to leading order in $1/N$, we find

$$\begin{aligned}
\mathbf{E}\Big\{\tilde{\mathbf{J}}^n_{ij}\Big\}_{i\in X, j\in Y} &= \sum_{k_1,\ldots,k_{n-1}=1}^{N} \mathbf{E}\Big\{\tilde{\mathbf{J}}_{ik_{n-1}}\cdots\tilde{\mathbf{J}}_{k_2k_1}\tilde{\mathbf{J}}_{k_1j}\Big\}_{i\in X, j\in Y} \\
&= \sum_{k_1,\ldots,k_{n-1}=1}^{N} \mathbf{E}\Big\{\tilde{\mathbf{J}}_{ik_{n-1}}\Big\}\cdots\mathbf{E}\Big\{\tilde{\mathbf{J}}_{k_2k_1}\Big\}\mathbf{E}\Big\{\tilde{\mathbf{J}}_{k_1j}\Big\}_{i\in X, j\in Y} + \mathcal{O}(1/N^2) \\
&= \left(\sum_{k_{n-1}} \mathbf{E}\Big\{\tilde{\mathbf{J}}_{ik_{n-1}}\Big\}\right)\cdots\left(\sum_{k_1} \mathbf{E}\Big\{\tilde{\mathbf{J}}_{k_2k_1}\Big\}\right)\mathbf{E}\Big\{\tilde{\mathbf{J}}_{k_1j}\Big\}_{i\in X, j\in Y} + \mathcal{O}(1/N^2) \\
&= \tilde{\mu}^{n-1}\frac{\tilde{\mu}_Y}{N_Y} + \mathcal{O}(1/N^2).
\end{aligned} \tag{9}$$

where in factoring the sum in the second-to-last equality, we used that the expectation of a single connection depends only on the pre-synaptic cell (the second index). Similarly, we find that

$$\mathbf{E}\{\mathbf{J}_{ij}^{m*}\}_{i\in X, j\in Y} = \tilde{\mu}^{(m-1)*}\frac{\tilde{\mu}_X^*}{N_X} + \mathcal{O}(1/N^2).$$

1.2.4 Case 4: $3 \leq n+m \ll N$, and $n, m \neq 0$

These values of n, m correspond to direct and indirect common input motifs in the network. A sufficient condition for the terms to factor as in Eq. (5) is the independence of the matrix entries. This will certainly be true if the entries come from different rows of $\tilde{\mathbf{J}}$, i.e., if all of the second indices in Eq. (5) have distinct second entries:

$$i \neq k_{n-1} \neq k_{n-2} \neq \cdots \neq k_1 \neq l_1 \neq \cdots \neq l_{m-1} \neq j.$$

Note that since k_0 does not appear as a row index, we do not need to condition on k_0 (this leads to the multiplicative factor N below). As previously mentioned, we also can not factor the term involving k_0 in Eq. (5) as, due to Dale's principle, each neuron makes only positive (excitatory) or negative (inhibitory) synaptic interactions, so that the values $\tilde{\mathbf{J}}_{k_1k_0}$ and $\tilde{\mathbf{J}}_{l_1k_0}$ are not approximately independent in the same manner as other connections. The problem, then, is to select $n+m-2$ indices distinctly, when they are being chosen from $\{1,\ldots,N\}\backslash\{i,j\}$. So long as $n+m \leq N$, there are $P(N-2, n+m-2)$ ways to do this, where $P(x,y)$ stands for the number of permutations of y elements chosen from a pool of x without replacement. In addition, if $n+m-1 \ll N$, then $NP(N-2, n+m-2) \sim N^{n+m-1} + \mathcal{O}(N^{n+m-2})$. It follows that, out of the N^{n+m-1} terms comprising this sum, only $\mathcal{O}(N^{n+m-2})$ do not cancel. Since each term in the sum is scaled by $1/N^{n+m}$, this implies that the error is only $\mathcal{O}(1/N^2)$.

In this case, we then have by the approximate independence of connections when $n, m \ll N$

$$\begin{aligned}
\mathbf{E}\Big\{[\tilde{\mathbf{J}}^n(\tilde{\mathbf{J}}^*)^m]_{ij}\Big\}_{i\in X, j\in Y} &= \sum_{k_0,\ldots,k_{n-1},l_1,\ldots,l_{m-1}=1}^{N} \mathbf{E}\Big\{\tilde{\mathbf{J}}_{ik_{n-1}}\tilde{\mathbf{J}}_{k_{n-1}k_{n-2}}\cdots\tilde{\mathbf{J}}_{k_1k_0}\tilde{\mathbf{J}}_{l_1k_0}^*\cdots\tilde{\mathbf{J}}_{jl_{m-1}}^*\Big\} \\
&= \sum_{k_0,\ldots,k_{n-1},l_1,\ldots,l_{m-1}=1}^{N} \mathbf{E}\Big\{\tilde{\mathbf{J}}_{ik_{n-1}}\Big\}\mathbf{E}\Big\{\tilde{\mathbf{J}}_{k_{n-1}k_{n-2}}\Big\}\cdots\mathbf{E}\Big\{\tilde{\mathbf{J}}_{k_1k_0}\tilde{\mathbf{J}}_{l_1k_0}^*\Big\}\cdots\mathbf{E}\Big\{\tilde{\mathbf{J}}_{jl_{m-1}}^*\Big\} + \mathcal{O}(1/N^2) \\
&= \left(\sum_{k_{n-1}}\mathbf{E}\Big\{\tilde{\mathbf{J}}_{ik_{n-1}}\Big\}\right)\left(\sum_{k_{n-2}}\mathbf{E}\Big\{\tilde{\mathbf{J}}_{k_{n-1}k_{n-2}}\Big\}\right)\cdots\left(\sum_{k_0}\mathbf{E}\Big\{\tilde{\mathbf{J}}_{k_1k_0}\tilde{\mathbf{J}}_{l_1k_0}^*\Big\}\right)\cdots \\
&\quad \cdots\left(\sum_{l_{m-1}}\mathbf{E}\Big\{\tilde{\mathbf{J}}_{jl_{m-1}}^*\Big\}\right) + \mathcal{O}(1/N^2) \\
&= \tilde{\mu}^{n-1}\tilde{\mu}^{(m-1)*}\tilde{\mu}_c + \mathcal{O}(1/N^2),
\end{aligned} \tag{10}$$

Note that the approximation in Eq. (10) is not exactly valid if $i=j$, as the first and last terms are no longer independent. However, the difference will be provably only $\mathcal{O}(1/N^2)$ in this case, so the approximation remains valid.

1.2.5 Case 5: n, m not significantly smaller than N

Even though the above approximations are no longer valid when n, m are not significantly smaller than N, the size of these terms will decay exponentially with an upper bound $\sim r^{n+m}$, where r is the radius of the eigenvalue spectrum of the matrix $\tilde{A}\tilde{\mathbf{J}}$ [1]. The implicit assumption we make is that by the time the above approximations start to fail, n, m are large enough so as to make those terms insignificant. This radius generally will vary with ω, so we assume the existence of a uniform bound less than $r = 1$ for all ω. From numerical investigation his seems to be a reasonable assumption to make in general for the networks we consider.

1.3 Results

Applying Eqs. (6–10) to Eq. (4) yields the following expression when $i \neq j$

$$\begin{aligned}\mathbf{E}\Big\{\tilde{\mathbf{C}}_{ij}\Big\}_{i\in X, j\in Y} &= \tilde{C}^0 \left[\left(\sum_{n=1}^{\infty} \tilde{A}^n \tilde{\mu}^{n-1}\right)\frac{\tilde{\mu}_Y}{N_Y} + \left(\sum_{m=1}^{\infty} \tilde{A}^{m*} \tilde{\mu}^{(m-1)*}\right)\frac{\tilde{\mu}_X^*}{N_X}\right.\\ &\qquad \left. + \left(\sum_{n,m=1}^{\infty} (\tilde{A}^n \tilde{\mu}^{n-1})(\tilde{A}^{m*}\tilde{\mu}^{(m-1)*})\right)\tilde{\mu}_c\right] + \mathcal{O}(1/N^2) \\ &= \tilde{C}^0 \left[\left(\frac{\tilde{A}}{1-\tilde{A}\tilde{\mu}}\right)\frac{\tilde{\mu}_Y}{N_Y} + \left(\frac{\tilde{A}}{1-\tilde{A}\tilde{\mu}}\right)^*\frac{\tilde{\mu}_X^*}{N_X} + \left|\frac{\tilde{A}}{1-\tilde{A}\tilde{\mu}}\right|^2 \tilde{\mu}_c\right] + \mathcal{O}(1/N^2).\end{aligned} \tag{11}$$

When $i = j$, an additional correction term enters at second order due to the fact that in the random network, when computing the correlation between a neuron and itself, it does not share a "random" amount of common input with itself, but a deterministic amount given by the in-degree and strength of connections. Hence, the terms corresponding to common input are scaled upwards in strength as seen in Eq. (8). Hence, when $i = j$, we have

$$\begin{aligned}\mathbf{E}\Big\{\tilde{\mathbf{C}}_{ii}\Big\}_{i\in X} &= \tilde{C}^0 \left[\left(\sum_{n=1}^{\infty} \tilde{A}^n \tilde{\mu}^{n-1}\right)\frac{\tilde{\mu}_X}{N_X} + \left(\sum_{m=1}^{\infty} \tilde{A}^{m*} \tilde{\mu}^{(m-1)*}\right)\frac{\tilde{\mu}_X^*}{N_X} + |\tilde{A}|^2\frac{\tilde{\mu}_c}{p}\right.\\ &\qquad \left. + \left(\sum_{\substack{n,m=1\\ n+m\geq 3}}^{\infty} (\tilde{A}^n \tilde{\mu}^{n-1})(\tilde{A}^{m*}\tilde{\mu}^{(m-1)*})\right)\tilde{\mu}_c\right] + \mathcal{O}(1/N^2) \\ &= \tilde{C}^0 \left[1 + \left(\frac{\tilde{A}}{1-\tilde{A}\tilde{\mu}}\right)\frac{\tilde{\mu}_X}{N_X} + \left(\frac{\tilde{A}}{1-\tilde{A}\tilde{\mu}}\right)^*\frac{\tilde{\mu}_X^*}{N_X}\right.\\ &\qquad \left. + \left|\frac{\tilde{A}}{1-\tilde{A}\tilde{\mu}}\right|^2 \tilde{\mu}_c + |\tilde{A}|^2\tilde{\mu}_c\left(\frac{1-p}{p}\right)\right] + \mathcal{O}(1/N^2)\end{aligned} \tag{12}$$

2 First order conditioned averages in the random network

We may also approximate expected correlations for a pair of cells of a specified type conditioned on their first-order (direct) connectivity in the random, fixed in-degree network to $\mathcal{O}(1/N^2)$. The calculations follow similar to the proofs for cell-type averages above with minor modifications.

2.1 Leading order effects of conditioning

For a first example example, suppose are interested in the expected value of a single connection originating from cell i conditioned on two excitatory connections $\mathbf{J}_{ij}, \mathbf{J}_{ji}$ (so the indices i, j refer to excitatory cells we will assume to be distinct). We do not specify in our conditioning whether the connections are present or not — only that we know their value. We may solve for the expectation of an outgoing connection from cell i (with an identical conclusion holding for cell j):

$$\begin{aligned}
\mathbf{E}\Big\{\tilde{\mathbf{J}}_{ai}|\tilde{\mathbf{J}}_{ij}, \tilde{\mathbf{J}}_{ji}\Big\}_{i,j\in E,a} &= \frac{1}{N}\sum_a \mathbf{E}\Big\{\tilde{\mathbf{J}}_{ai}|\tilde{\mathbf{J}}_{ij}, \tilde{\mathbf{J}}_{ji}\Big\}_{i,j\in E} \\
&= \begin{pmatrix}\text{Probability that cell} \\ a \text{ is excitatory}\end{pmatrix}\begin{pmatrix}\text{Expected value of the connection} \\ \text{if } a \text{ is excitatory}\end{pmatrix} \\
&\quad + \begin{pmatrix}\text{Probability that cell} \\ a \text{ is inhibitory}\end{pmatrix}\begin{pmatrix}\text{Expected value of the connection} \\ \text{if } a \text{ is inhibitory}\end{pmatrix} \\
&= f_E\left(\frac{G_E}{pN_E}\tilde{F}_E\right)\left(p + \frac{1-\delta_{\tilde{\mathbf{J}}_{ji},0} - p}{N_E}\right) + f_I\left(\frac{G_E}{pN_E}\tilde{F}_E\right)p \\
&= \frac{\tilde{\mu}_E}{N_E} + \frac{(1-\delta_{\tilde{\mathbf{J}}_{ji},0} - p)G_E}{pN^2} \\
&= \frac{\tilde{\mu}_E}{N_E} + \mathcal{O}(1/N^2)
\end{aligned} \tag{13}$$

where the subscript a denotes an average over all possible values of the index a, and the term $\Big(p + (1-\delta_{\tilde{\mathbf{J}}_{ji},0} - p)/N_E\Big)$ represents the fraction of connections cell i will make on to other excitatory cells which are expected to be present. This will be greater or less than p, the connection probability, depending on whether $\tilde{\mathbf{J}}_{ji}$ is on or off. If it is on, for example, the expected number of outgoing connections to excitatory cells which will be on is $1 + p(N_E - 1)$, as unconditioned outgoing connections (i.e., $\tilde{\mathbf{J}}_{ki}$ for $k \neq j$) are multiples of Bernoulli random variables. Note that the first-order conditioned expected value coincides with the unconditioned expected value to leading order in $1/N$.

We also have that

$$\begin{aligned}
\sum_b \mathbf{E}\Big\{\tilde{\mathbf{J}}_{ab}|\tilde{\mathbf{J}}_{ij}, \tilde{\mathbf{J}}_{ji}\Big\}_{i,j\in E} &= \mathbf{E}\left\{\left(\sum_b \tilde{\mathbf{J}}_{ab}\right)\middle|\, \tilde{\mathbf{J}}_{ij}, \tilde{\mathbf{J}}_{ji}\right\}_{i,j\in E} = \tilde{\mu} \quad \text{and} \\
\sum_{b\in X} \mathbf{E}\Big\{\tilde{\mathbf{J}}_{ab}|\tilde{\mathbf{J}}_{ij}, \tilde{\mathbf{J}}_{ji}\Big\}_{i,j\in E} &= \mathbf{E}\left\{\left(\sum_{b\in X} \tilde{\mathbf{J}}_{ab}\right)\middle|\, \tilde{\mathbf{J}}_{ij}, \tilde{\mathbf{J}}_{ji}\right\}_{i,j\in E} = \tilde{\mu}_X
\end{aligned} \tag{14}$$

for any a, agreeing with the values of the same quantities found in the absence of conditioning on connection values above. We have fixed the in degree so that the fact that certain connections are present or absent has no bearing on the expected input to a particular cell.

We may also solve for the value of $\tilde{\mu}_c$ when we condition on the first order connectivity of two

excitatory cells:

$$
\begin{aligned}
\tilde{\mu}_{EE,c} &= \mathbf{E}\Big\{[\tilde{\mathbf{J}}\tilde{\mathbf{J}}^T]_{ij}|\tilde{\mathbf{J}}_{ij},\tilde{\mathbf{J}}_{ji}\Big\}_{i\in E, j\in E} \\
&= \sum_{k=1}^{N}\mathbf{E}\Big\{\tilde{\mathbf{J}}_{ik}\tilde{\mathbf{J}}_{jk}|\tilde{\mathbf{J}}_{ij},\tilde{\mathbf{J}}_{ji}\Big\} \\
&= \sum_{k\neq i,j}\mathbf{E}\Big\{\tilde{\mathbf{J}}_{ik}\tilde{\mathbf{J}}_{jk}|\tilde{\mathbf{J}}_{ij},\tilde{\mathbf{J}}_{ji}\Big\} + \mathbf{E}\Big\{\tilde{\mathbf{J}}_{ii}\tilde{\mathbf{J}}_{ji}|\tilde{\mathbf{J}}_{ij},\tilde{\mathbf{J}}_{ji}\Big\} + \mathbf{E}\Big\{\tilde{\mathbf{J}}_{ij}\tilde{\mathbf{J}}_{jj}|\tilde{\mathbf{J}}_{ij},\tilde{\mathbf{J}}_{ji}\Big\} \\
&= \sum_{k\neq i,j}\mathbf{E}\Big\{\tilde{\mathbf{J}}_{ik}\tilde{\mathbf{J}}_{jk}|\tilde{\mathbf{J}}_{ij},\tilde{\mathbf{J}}_{ji}\Big\} + \tilde{\mathbf{J}}_{ji}\mathbf{E}\Big\{\tilde{\mathbf{J}}_{ii}|\tilde{\mathbf{J}}_{ij},\tilde{\mathbf{J}}_{ji}\Big\} + \tilde{\mathbf{J}}_{ij}\mathbf{E}\Big\{\tilde{\mathbf{J}}_{jj}|\tilde{\mathbf{J}}_{ij},\tilde{\mathbf{J}}_{ji}\Big\} \\
&= (N_E-2)\left(\frac{G_E}{pN_E}\tilde{F}_E\right)^2 p_i p_j + N_I\left(-\frac{G_I}{pN_I}\tilde{F}_I\right)^2 p^2 \\
&\quad + \tilde{\mathbf{J}}_{ij}\left(\frac{G_E}{pN_E}\tilde{F}_E\right)p_i + \tilde{\mathbf{J}}_{ji}\left(\frac{G_E}{pN_E}\tilde{F}_E\right)p_j
\end{aligned}
$$

where we define p_i to be the probability that an excitatory connection $k \to i$ exists when $k \neq j$ conditioned on the value of $\tilde{\mathbf{J}}_{ij}$.This may be explicitly calculated as

$$
p_i = \begin{cases} \frac{pN_E-1}{N_E-1} & \text{if} \quad \tilde{\mathbf{J}}_{ij} \neq 0 \\ \frac{pN_E}{N_E-1} & \text{if} \quad \tilde{\mathbf{J}}_{ij} = 0 \end{cases}
$$

with identical calculations holding for p_j for the probability of an excitatory connection $k \to j$ when $k \neq i$. In either case, it is easy to see that $p_i = p + \mathcal{O}(1/N)$. Using this, we find that

$$
\tilde{\mu}_{EE,c} = \tilde{\mu}_c + \mathcal{O}(1/N^2), \tag{15}
$$

so that this value is again unchanged, to leading order in $1/N$, from the unconditioned case.

The previous results regarding the approximate independence of connections in this network still hold. Equations (13-15) reveal that that, to leading order in $1/N$, there will be no change to the expectations of terms higher than first order as a result of conditioning on the values of two excitatory connections. Hence, the only difference occurs at first order, where we are replacing average connection values with known connection values (see Eq. (16) below). Also, note that we've shown only the calculations for the excitatory-excitatory first order conditioned averages. However, there is nothing unique about this case - the inhibitory-inhibitory and excitatory-inhibitory cases may be solved likewise.

2.2 Results

If we condition on the connectivity and cell-type of two distinct cells in the random, fixed in-degree network and average over pairs of this type, we find that

$$
\begin{aligned}
\mathbf{E}\Big\{\tilde{\mathbf{C}}_{ij}|\tilde{\mathbf{J}}_{ij},\tilde{\mathbf{J}}_{ji}\Big\}_{i\in X, j\in Y} &= \tilde{C}^0\Bigg[\tilde{A}\tilde{\mathbf{J}}_{ij} + \tilde{A}^*\tilde{\mathbf{J}}_{ji} + \left(\sum_{n=2}^{\infty}\tilde{A}^n\tilde{\mu}^{n-1}\right)\frac{\tilde{\mu}_Y}{N_Y} + \left(\sum_{m=2}^{\infty}\tilde{A}^{m*}\tilde{\mu}^{(m-1)*}\right)\frac{\tilde{\mu}_X^*}{N_X} \\
&+ \left(\sum_{n,m=1}^{\infty}(\tilde{A}^n\tilde{\mu}^{n-1})(\tilde{A}^{m*}\tilde{\mu}^{(m-1)*})\right)\tilde{\mu}_c\Bigg] + \mathcal{O}(1/N^2)
\end{aligned} \tag{16}
$$

3 Linear response for conductance-based model neurons

A more realistic model of neural activity is an IF neuron receiving noisy inputs and connecting to other neurons via conductances [2]. Consider a network of N such model neurons with membrane potentials evolving according to

$$\begin{aligned} C_{m,i}\dot{v}_i &= -g_{L,i}(v_i - E_{L,i}) + \psi(v_i) + (f_{E,i}(t) + g_{E,i}(t))(E_E - V) + (f_{I,i}(t) + g_{I,i}(t))(E_I - V) \\ g_{E,i} &= C_{m,i}a_{E,i}S_{E,i}(t) \\ g_{I,i} &= C_{m,i}a_{I,i}S_{I,i}(t) \end{aligned} \tag{17}$$

where $S_{E,i}(t), S_{I,i}(t)$ are independent Poisson spiking processes of rates $\lambda_{E,i}, \lambda_{I,i}$, respectively, which account for inputs not explicitly modeled, $a_{E,i}, a_{I,i}$ set the impact of a spike from these processes, E_E, E_I are reversal potentials, and $f_{E,i}, f_{I,i}$ account for excitatory and inhibitory synaptic conductance inputs from the modeled network. We again model the time course of synaptic coupling within the network with alpha functions, and coupling strengths will now denote the total conductance delivered (See main text).

We will adopt the usual diffusion approximation to the Poisson conductance inputs [3–7]. Under this approximation, the excitatory conductance is

$$g_{E,i} = C_{m,i}(\mu_{E,i} + \sigma_{E,i}\xi_{E,i}(t)),$$

where

$$\mu_{E,i} = b_{E,i}\lambda_{E,i}, \qquad \sigma_{E,i} = b_{E,i}\sqrt{\lambda_{E,i}}, \tag{18}$$

and $b_{E,i} = 1 - e^{-a_{E,i}}$ (we use the Stratonovich interpretation of the terms $V\delta(t - t_0)$ on the right hand side of Eq. (17), see Stratonovich [8]). $\xi_{E,i}$ is a Gaussian white noise process with $\langle \xi_{E,i}(t)\rangle = 0$ and $\langle \xi_{E,i}(t)\xi_{E,i}(t')\rangle = \delta(t - t')$. This approximation is valid in the limit

$$\lambda_{E,i} \gg 1 \qquad a_{E,i} \ll 1.$$

Hence, the diffusion approximation of the system in Eq. (17) is

$$\begin{aligned} \tau_i\dot{v}_i = -(v_i - E_{L,i} - \psi(v_i)) + \left[f_{E,i}(t)/g_{L,i} + \tau_i(\mu_{E,i} + \sigma_{E,i}\xi_{E,i}(t))\right](E_E - V) + \dots \\ \left[f_{I,i}(t)/g_{L,i} + \tau_i(\mu_{I,i} + \sigma_{I,i}\xi_{I,i}(t))\right](E_I - V) \end{aligned} \tag{19}$$

The situation is not complicated much versus the case of current-based inputs and noise considered in the main text. With regards to the behavior of the single cell, by defining an effective membrane time constant, rest potential and noise intensity

$$\tau_i^{eff} = \frac{\tau_i}{1 + g_{L,i}^{-1}[\langle f_{E,i}\rangle + \langle f_{I,i}\rangle] + \tau_i[\mu_{E,i} + \mu_{I,i}]}$$

$$E_{L,i}^{eff} = \frac{E_{L,i} + E_E(g_{L,i}^{-1}\langle f_{E,i}\rangle + \tau_i\mu_{E,i}) + E_I(g_{L,i}^{-1}\langle f_{I,i}\rangle + \tau_i\mu_{I,i})}{1 + g_{L,i}^{-1}[\langle f_{E,i}\rangle + \langle f_{I,i}\rangle] + \tau_i[\mu_{E,i} + \mu_{I,i}]}$$

$$\sigma_i^{eff}(v) = \sqrt{\tau_i^{eff}\left[\sigma_{E,i}^2(E_E - v)^2 + \sigma_{I,i}^2(E_I - v)^2\right]}$$

and rewriting Eq. (19), we have

$$
\begin{aligned}
\tau_i^{eff}\dot{v}_i = -(v_i - E_{L,i}^{eff} - \psi(v_i)) + (f_{E,i}(t) - \langle f_{E,i}\rangle)(E_E - v_i) + \ldots \\
(f_{I,i}(t) - \langle f_{I,i}\rangle)(E_I - v_i) + \sqrt{2\tau_i^{eff}}\sigma_i^{eff}(v_i)\xi_i(t).
\end{aligned} \tag{20}
$$

This is known as the effective time constant approximation [7, 9].

The main differences between the conductance-based case and the current-based case are that fluctuations due to input from the modeled network now occur in conductances, and that the noise variance now depends on the membrane potential . Also, the fluctuations of the excitatory and inhibitory inputs must be considered separately (whereas in the current-driven case, the two could be lumped together and we considered only the response to fluctuations in a single input current). This necessitates the need for slightly different linear response. However, making use of the framework established by Richardson [10], one can easily derive simple boundary value problems which can be solved for the firing rate, uncoupled power spectrum, and firing rate response for modulation of mean conductances of a cell receiving. Each cell now has two linear response functions - one for response to modulation of the mean excitatory conductance ($A_{E,i}$) and one for response to modulation of the mean inhibitory conductance ($A_{I,i}$). The kernel $A_{E,i}$ was derived in analytical form by Richardson [11] for a very similar case.

Using the same iterative approach as in the main text, we can arrive at an approximation to correlations in a recurrent network of conductance-coupled excitatory and inhibitory neurons:

$$
\tilde{\mathbf{C}}(\omega) = \lim_{n\to\infty}\tilde{\mathbf{C}}^n(\omega) = (\mathbf{I} - \tilde{\mathbf{K}}(\omega))^{-1}\tilde{\mathbf{C}}^0(\omega)(\mathbf{I} - \tilde{\mathbf{K}}^*(\omega))^{-1} \tag{21}
$$

where now we have

$$
[\tilde{\mathbf{K}}_{ij}]_{j\in X} = \mathbf{W}_{ij}\tilde{A}_{X,i}\tilde{J}_{X,j} \tag{22}
$$

Note that the necessity of two linear response functions does not cause any complication when compared with the current-driven case considered in the main text. Excitation and inhibition already had different synaptic kernels (even if only in sign). Excitatory response functions are applied to excitatory cells, and likewise with inhibitory, so that the difference in excitatory and inhibitory response functions reduces to an effective difference in coupling filters for inputs coming from the two cell classes. We compare the results obtained from Eq. (21) for conductance-based neurons with those obtained from Monte Carlo estimation of cross-correlations in Figure S1. We confirm the match only for a small circuit here — further exploration would be required in order to fully explore the accuracy of approximations in large networks.

4 The effect of autaptic connections in the all–to–all network

As stated in the main text, the difference in cross- and auto-correlations is minor in the all–to-all network when autaptic connections are included or excluded (See Figure S2). The auto-correlations show a larger absolute difference because omitting autapses corresponds to omitting the first order term representing the effects of direct connections between a cell and itself. However, in this particular network, the zeroth order terms dominated the auto-correlations, decreasing the relative impact of the lost first order term, while in the cross-correlations, omission of autapses induced an, at most, order $1/N$ difference in each term. As the correlations themselves are already order $1/N$, the difference is order $1/N^2$, giving the small relative error we see.

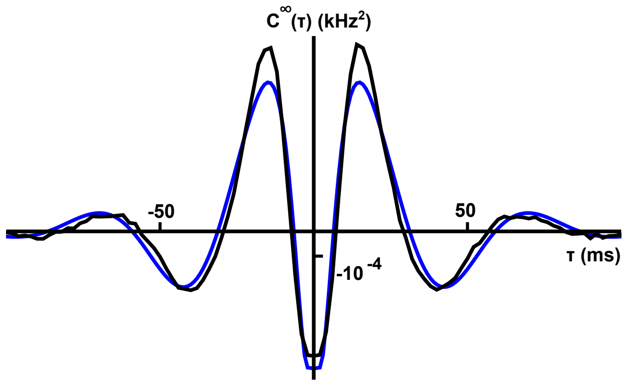

Figure S1. Estimating correlations via linear response in the presence of conductance coupling. Comparison of the theoretical prediction with the numerically computed cross-correlation between two reciprocally conductance-coupled excitatory cells. Parameters were $g_L = 5.7$ nS, $C_m = 114$ pF, $E_L = -75$ mV, $E_E = 0$ mV, $\lambda_E = 5$ kHz, $a_E = 0.004$, $E_I = -90mV$, $\lambda_I = a_I = 0$, $V_T = -54$ mV, $\Delta_T = 1$ mV, $V_r = -57$ mV, $v_{th} = 20$ mV, $\tau_{ref} = 2$ ms.

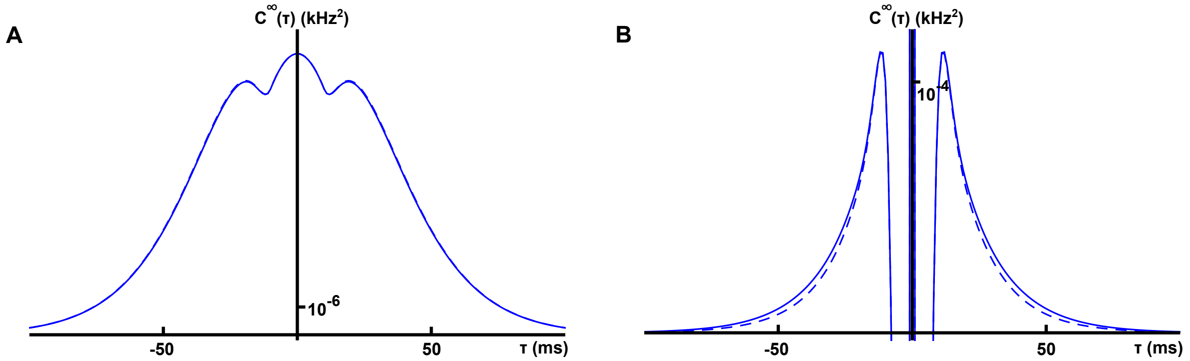

Figure S2. The effect of autaptic connections in the all–to–all network. A. Comparison of theoretical predictions of the cross-correlations in an all–to–all network of size $N = 100$ with (solid) and without (dashed) autaptic connections. Relative L^2 error ≈ 0.0043. **B.** Same as panel A, for auto-correlations. Relative L^2 error ≈ 0.0016. All parameters are the same as in the balanced all–to–all network of the main text.

5 Limits of the Theory

We have performed a thorough investigation of the limits of the theory. We started by investigating when the theory fails to accurately predict a PSTH for a single cell. The idea is that if the theory does not give accurate predictions for a single cell, then it should fail in a network. We then investigated how well the theory predicts correlations in a complete network. The results are described in the following text which has been added to the Supplementary Information:

Here we explore the limitations of the linear response theory. First, we examined the limits of linear response theory in predicting time-dependent firing activity (the peri-stimulus time histogram, or PSTH) in the balanced, all–to–all network considered in Figure 6 of the main text. We generated 100 realizations of Poisson spiking processes, each 2s in duration. We designated 80 to be excitatory and 20 to be inhibitory, to emulate the input received by a cell in the all–to–all network. These point processes were convolved with weighted alpha functions (see Eq. (2) in the

main text), and used to drive an EIF cell which was also receiving a white, fluctuating background input. As in Figure 1 of the main text, we averaged over realizations of background noise to obtain the time-dependent firing intensity of this post-synaptic cell. We quantified the agreement between the result obtained by numerical simulation and the approximation obtained using linear response theory by computing the Pearson correlation coefficient between the two. This was performed over a range of input noise intensities and connection weights to obtain Figure S3A. In agreement with the expectation that noise linearizes responses and improves the linear response approximation, the agreement is best when noise is strong and connections weak, and worst for weak noise and strong connections. Notably, for a large range of parameters, the linear response prediction of the PSTH was quite good, with Pearson correlation coefficients above 80%.

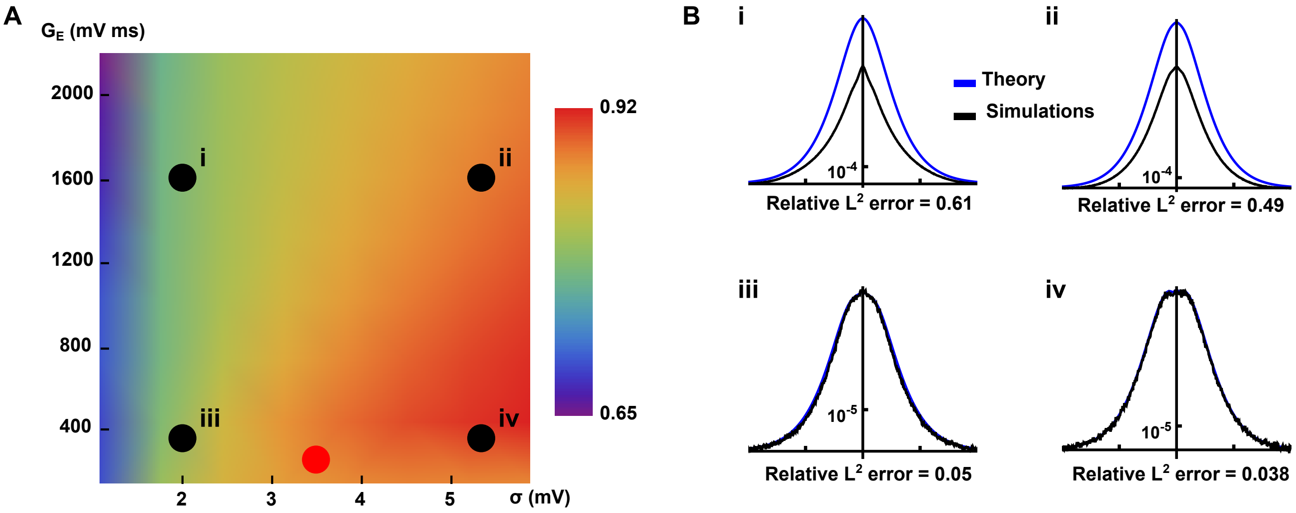

Figure S3. Evaluating the accuracy of linear response predictions of the PSTH and of cross-correlations. A. The Pearson correlation coeffecient between the time-dependent firing activity (PSTH) calculated from Monte Carlo simulations and linear response theory for an array of σ (noise intensity) and G_E (total excitatory/inhibitory connection weight) values for a single EIF neuron receiving 80 excitatory and 20 inhibitory Poisson inputs at 17 Hz, convolved with alpha synaptic kernels (see Eq. (2) in the manuscript). These inputs emulated the total drive received by a neuron in the precisely balanced, all–to–all network considered in Figure 6 of the main text. A higher correlation indicates a closer match between simulations and theory. The red dot indicates the parameters used for Figure 6. **B.** The cross-correlation function between two excitatory cells in the precisely balanced, all–to–all network from Monte Carlo simulations and linear response theory at four points in (σ, G_E) space, indicated by the black dots in panel A.

Approximating time-dependent firing activity (PSTH) is only a part of approximating network correlations. Different network effects may limit the accuracy of the approximation given by Eq. (15). We therefore compared numerically obtained cross-correlation functions with those given by Eq. (22) at four points (indicated by black dots in Figure S3A). The results shown in Figure S3B indicate that the trends in cross-correlations and PSTH approximation errors are similar. In particular, for connection strengths still stronger than those used in the paper (red dot), the match between theory and simulations is excellent at both noise levels tested. However, in networks, large connection strengths (over 10x those used in Figure 6 of the main text) can cause the approximation of cross-correlations to be worse than what would be expected from single cell activity. In

particular, note that the relative L^2 error[1] between theoretical and numerical predictions was much lower for cases (iii), (iv) when connection strengths were weak. Nevertheless, in the case of strong connectivity (examples (i), (ii)), the relative error was significantly reduced in the high noise case (ii).

Another setting in which the linear response theory might be expected to fail is the case of low firing rates. To examine the effect, we revisit the FFI microcircuit considered in the main text. We varied the effective rest potential $E_{L,i} + E_i$ of the downstream excitatory cell E_2, while fixing the remaining parameters. The theoretically and numerically obtained cross-correlations are shown in Figure S4 at three values used for the effective rest potential.

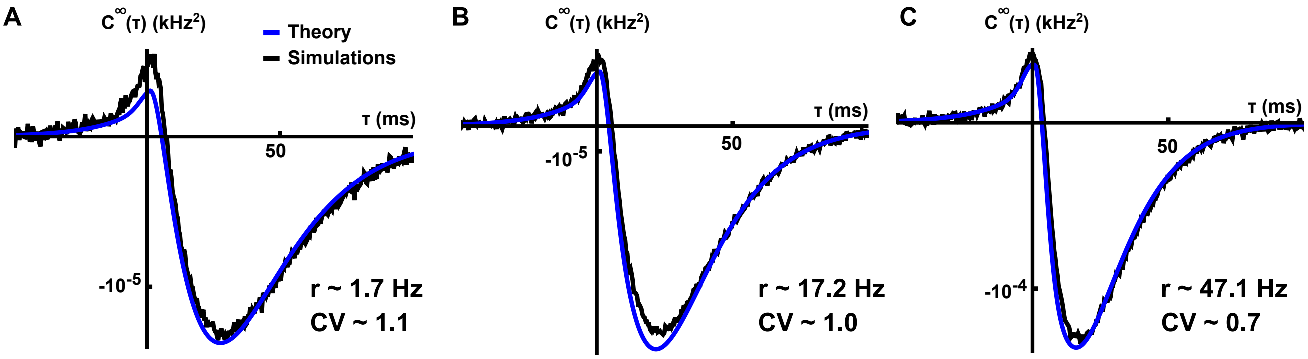

Figure S4. The performance of linear response theory in various firing regimes. For the feed-forward inhibitory microcircuit considered in the manuscript (see Figure 3 of the main text), we varied the effective rest potential $E_{L,i} + E_i$ (see Eq. (1) in the main text) to the cell E_2 between three values — **A.** $E_{L,i} + E_i = -59$ mV, **B.** $E_{L,i} + E_i = -54$ mV (this is the value used in the example in the main text), **C.** $E_{L,i} + E_i = -49$ mV. Inset is the firing rate and the coefficient of variation of the interspike interval distribution of cell E_2.

For comparison, we have also included the CV of the ISI distribution for the cell E2. The three chosen parameter values lead to three quantitatively and qualitatively different firing behaviors. In particular, when the effective rest potential is -59 mV, firing is spontaneous, but rare (rate $\sim$ 1.7 Hz, CV $\sim$ 1.1). Although the error does seem to increase as firing rates decreases, the theory still performs well even for very low-rate spiking. We suspect that part of this error is due to the propensity of linear response theory to predict negative (nonphysical) firing rates when rates are low but connections are strong.

References

[1] Pernice V, Staude B, Cardanobile S, Rotter S (2011) How structure determines correlations in neuronal networks. PLoS Comput Biol 7: e1002059.

[2] Destexhe A (1997) Conductance-based integrate-and-fire models. Neural Comput 9: 503–514.

[3] Ricciardi L, Sacerdote L (1979) The ornstein-uhlenbeck process as a model for neuronal activity. Biol Cybern 35: 1–9.

[1] The relative L^2 error between the linear response-predicted correlation C^∞ and the Monte Carlo prediction C was defined as $error = ||C^\infty - C||_2/||C||_2$.

[4] Risken H (1996) The Fokker-Planck equation: Methods of solution and applications. Berlin: Springer Verlag, 488 pp.

[5] Gardiner C (2009) Handbook of Stochastic Methods for Physics, Chemistry and the Natural Sciences. Berlin: Springer-Verlag, 464 pp.

[6] Renart A, Brunel N, Wang X (2004) Mean-field theory of irregularly spiking neuronal populations and working memory in recurrent cortical networks. In: Feng J, editor, Computational Neuroscience: A Comprehensive Approach, Boca Raton: CRC Press. pp. 431–490.

[7] Burkitt A (2006) A review of the integrate-and-fire neuron model: I. homogeneous synaptic input. Biol Cybern 95: 1–19.

[8] Stratonovich R, Silverman R (1967) Topics in the Theory of Random Noise, volume 2. New York: Gordon and Breach, 610 pp.

[9] Johannesma P (1968) Diffusion models for the stochastic activity of neurons. Neural networks : 116–144.

[10] Richardson M (2007) Firing-rate response of linear and nonlinear integrate-and-fire neurons to modulated current-based and conductance-based synaptic drive. Phys Rev E 76: 021919.

[11] Richardson M (2009) Dynamics of populations and networks of neurons with voltage-activated and calcium-activated currents. Phys Rev E 80: 021928.
